# Bioactive Potential of Wild Plants from Gardunha Mountain: Phytochemical Characterization and Biological Activities

**DOI:** 10.3390/molecules30193876

**Published:** 2025-09-25

**Authors:** Alexandra Coimbra, Eugenia Gallardo, Ângelo Luís, Pedro Dinis Gaspar, Susana Ferreira, Ana Paula Duarte

**Affiliations:** 1 RISE-Health, Department of Medical Sciences, Faculty of Health Sciences, University of Beira Interior, Av. Infante D. Henrique, 6200-506 Covilhã, Portugal; alexandra.coimbra@ubi.pt (A.C.); egallardo@fcsaude.ubi.pt (E.G.); angelo.luis@ubi.pt (Â.L.); susana.ferreira@fcsaude.ubi.pt (S.F.); 2 Laboratório de Fármaco-Toxicologia, UBIMedical, Universidade da Beira Interior, Estrada Municipal 506, 6200-284 Covilhã, Portugal; 3 Grupo de Investigação Sobre Problemas Relacionados Com Toxicofilias, Centro Académico Clínico das Beiras (CACB), Universidade da Beira Interior, Av. Infante D. Henrique, 6200-506 Covilhã, Portugal; 4 C-MAST—Center for Mechanical and Aerospace Science and Technologies, University of Beira Interior, 6201-001 Covilhã, Portugal; dinis@ubi.pt

**Keywords:** plant extracts, phytochemistry, anti-inflammatory activity, antioxidant activity, antimicrobial activity, cytotoxicity

## Abstract

The plants used in folk medicine have been increasingly studied to identify their bioactive properties. Therefore, this study aimed to assess the bioactivity of the hydroethanolic extracts of plants collected in Gardunha Mountain, Portugal. Seven abundant and representative wild plants were studied: *Cistus salviifolius*, *Clinopodium vulgare*, *Coincya monensis*, *Glandora prostrata*, *Helichrysum stoechas*, *Rubia peregrina*, and *Umbilicus rupestris*. The phytochemical composition of the extracts was determined by UHPLC-timsTOF-MS and by spectrophotometric methods. The antioxidant, in vitro anti-inflammatory and antimicrobial activity and the biocompatibility of the extracts were tested. The extracts were predominantly composed of flavonoids and phenolic acids, such as gallic acid, neochlorogenic acid and quercetin glycosides. The *C. salviifolius* extracts demonstrated very strong antioxidant activity related to scavenging free radicals (AAI = 2.84 and 2.93). Regarding antimicrobial activity, the *H. stoechas* extract exhibited inhibitory effects, particularly against Gram-positive bacteria and yeasts (MIC = 0.008–0.5 mg/mL). The *C. monensis*, *R. peregrina*, and *U. rupestris* extracts showed low cytotoxicity (viability > 70%) in the highest concentration tested. These findings highlight *C. salviifolius* and *H. stoechas* as promising sources of novel bioactive compounds, particularly antimicrobials in controlling microbial growth and promoting associated health benefits, and underscore the value of traditional medicinal plants as a guide for pharmacological studies.

## 1. Introduction

Since ancient times, plants have played a central role in traditional medicine and remain a primary source of therapeutic agents, especially in developing countries [1,2]. Due to several factors, including the growing demand for natural products, the rise in antibiotic resistance, and the ongoing search for new drugs, plants have increasingly attracted scientific interest, particularly those established in traditional medicine [3,4,5,6,7]. Among these, several plant families are well documented for their ethnobotanical applications and bioactive properties. Notable examples include: Boraginaceae (anticancer, anti-inflammatory, antimicrobial, antioxidant, and antidiabetic activities and neuroprotective effect) [8,9,10]; Brassicaceae (antioxidant, anti-inflammatory, antimicrobial, antiviral, antidiabetic, anticancer, and organ-protective activities) [11,12,13,14]; Cistaceae (antioxidant, antimicrobial, anti-inflammatory, and antidiabetic activity) [15,16]; Crassulaceae (anti-inflammatory, antiviral, antimicrobial, insecticidal, anticancer, and antioxidant activity and wound healing properties) [17,18,19]; Lamiaceae (antioxidant, anti-inflammatory, anticancer, antimicrobial, and cardioprotective activities and wound-healing and anti-ageing potential) [20,21,22,23,24]; and Rubiaceae (antimicrobial, analgesic, antidiabetic, anti-diarrheal, anti-pyretic, anti-inflammatory, antiviral, antiulcer, and antitumor activities) [25,26,27,28]. Different representative wild plants from these families can be found in the Gardunha Mountain, located in the central region of Portugal within the Iberian Massif, approximately 17 km west of Castelo Branco [29,30]. This mountain ridge separates the urban areas of Covilhã and Fundão. It is part of an extensive range of ridges stretching approximately 60 km in length, with a predominant NE-SW orientation [29,31]. The soil texture ranges from granular to silt loam, and the summers are extremely hot, with the region experiencing a water deficit of approximately 65%, where winters are mild and almost without snow cover [29,30]. These environmental conditions create a unique mountain ecosystem, where wild plants have adapted to local microclimates, soil composition, and seasonal water stress, factors that can influence their secondary metabolite profiles and biological activities. Despite their ecological and ethnobotanical relevance, many of these species remain poorly characterised. To address this gap, seven abundant and representative wild plants from Gardunha mountain were selected for further studies. Therefore, the present study aimed to evaluate their chemical composition and to provide a comprehensive understanding of their bioactive properties and biocompatibility, offering insights into their potential value for sustainable use and conservation.

## 2. Results and Discussion

A broad range of plants traditionally used in medicine have been studied by researchers for their health benefits, including antimicrobial and antioxidant activities [32]. Considering the potential of extracts, this work selected seven plants for study, based on their distribution in the Gardunha Mountain, traditional therapeutic uses, and their association with limited knowledge regarding their bioactivity.

### 2.1. Phytochemical Characterisation

The bioactive properties of the plant extracts are closely associated with their chemical composition [33]. Therefore, we started by determining the composition of the extracts, through a preliminary chemical analysis of their total phenolic and flavonoid content. The chemical composition of the extracts was obtained through the determination of total phenolic content using the Folin-Ciocalteu method, and the flavonoid content using the aluminium chloride method (Table 1).

The extract of *Glandora prostrata* (GP) exhibited the highest total phenolic content, followed by the extracts of *Clinopodium vulgare* (CV), and the stems and aerial parts of *Cistus salviifolius* (CSS and CSAP). Regarding total flavonoid content, the highest concentration was observed in the *Helichrysum stoechas* (HS) extract, followed by the extract obtained from the aerial parts of *C. salviifolius* (CSAP).

The hydroethanolic extract of *C. vulgare* showed a higher total phenolic content compared to ethanol, ethyl acetate, and acetone extracts studied in other works [34]. Among the analysed extracts by Todorova and collaborators (2016), the total aqueous extract of *C. vulgare* exhibited the highest total phenolic content, followed by the leaf aqueous extract, while the butanol extract showed the lowest total phenolic content [35]. Several studies have demonstrated that hydroethanolic extracts (e.g., 50–70% ethanol in water) exhibit significantly higher total phenolic content compared to extracts obtained with less polar solvents [36,37,38]. Therefore, the choice of plant part may account for the higher phenolic compound content observed when compared to the other plants.

Data on the extracts’ composition showed that *C. salviifolius* extracted with methanol acidified with 0.1% hydrochloric acid (HCl) was characterised by the presence of phenolic acids and their glycosides [39]. The ethanol and water extracts of the same plant had lower levels of total phenols (ranging from 46 to 50 mg GAE/g dry weight (DW)) and the total flavonoid content was lower than the values obtained in the present study but were expressed as catechin equivalents (mg CE/g DW), which may have an impact on the results obtained [40]. The ethanolic and aqueous extracts studied by Hitl and collaborators (2022) showed higher quantities of total phenols and lower total flavonoids as observed in the current investigation [41].

In line with this, when non-targeted metabolomic profiling of the extracts was analysed, it revealed a chemically diverse composition, predominantly comprising phenolic compounds, flavonoids, and coumarins. Compound annotation was performed using a three-tier approach: spectral library matching (SL), comparison with an in-house analyte list (AL), and elemental composition prediction via SmartFormula (SF), all processed using MetaboScape 7.0.1. Across all plant extracts, a total of 72 features were annotated with the highest level of confidence using the combined SL + AL + SF strategy, supported by accurate *m*/*z*, retention time, and collisional cross section (CCS) values. An additional 657 features were matched using the AL + SF combination, while 116 features were annotated via SL + SF. Furthermore, 2514 features were assigned based solely on SF, providing molecular formula predictions without spectral confirmation.

Comprehensive phytochemical screening of the studied plant extracts by UHPLC-timsTOF-MS enabled the tentative identification of numerous phenolic compounds, with notable variation across species and plant parts. Supplementary Figures S1 and S2 show the base peak chromatograms (BPC) of the samples acquired in positive and negative ionisation modes, respectively, providing complementary profiles of their chemical composition.

In *Cistus salviifolius*, the aerial parts showed a presence of flavonoids and phenolic acids, notably gallic acid, neochlorogenic acid, gallocatechin 3-*O*-gallate, and quercetin glycosides, including rutin and arabinoside forms. The stems also contained these compounds but with a slightly different profile, including kaempferol 3-*O*-glucosyl-rhamnosyl-galactoside and additional coumarins such as scopoletin and coumarin (Table S1).

The extract of *Clinopodium vulgare* featured abundant caffeic acid derivatives, rosmarinic acid, and flavonoid glycosides such as eriocitrin and luteolin derivatives. Noteworthy are also umbelliferone and esculin, suggesting coumarin-type compounds may be important contributors to its phytochemical fingerprint (Table S2).

*Coincya monensis* exhibited distinct profiles in its flowers and stems. Floral extracts were enriched in *p*-coumaric acid 4-*O*-glucoside, caffeic acid, apigenin 6,8-di-*C*-glucoside, and quercetin 3-*O*-arabinoside. The stems shared these compounds and additionally contained ellagic acid, dihydroquercetin 3-*O*-rhamnoside, and luteolin derivatives (Table S3).

In *Glandora prostrata*, the phytochemical profile was dominated by neochlorogenic acid, kaempferol 3-*O*-glucosyl-rhamnosyl-galactoside, and several flavones such as genistin and luteolin, as well as rosmarinic acid. Rhoifolin derivatives and umbelliferone were also identified (Table S4).

*Helichrysum stoechas* showed the presence of hydroxycinnamic acid esters, particularly 3-feruloylquinic acid and 3,4-dicaffeoylquinic acid, as well as chlorogenic and neochlorogenic acids. A range of flavonoids, including myricetin, quercetin, and luteolin glycosides, were also detected, along with scopoletin and usnic acid (Table S5).

The extract of *Rubia peregrina* was marked by a diverse flavonoid and phenolic acid profile, including di-*O*-methylbergenin, 3-feruloylquinic acid, quercetin and kaempferol derivatives, as well as anthraquinone-related structures suggested by the presence of emodin-like features (Table S6).

In *Umbilicus rupestris*, both flowers and leaves shared a core set of compounds such as gallocatechin, gallocatechin 3-*O*-gallate, quercetin 3-*O*-rutinoside, and rosmarinic acid. The flowers were additionally rich in esculetin, ellagic acid, and glycosylated flavones. The leaves contained sinapaldehyde, umbelliferone, and apigenin-*C*-glycosides, consistent with the plant’s use in topical formulations (Table S7).

Phenols and flavonoids are plant-derived compounds synthesised in response to various biotic and abiotic stresses. Their levels can vary depending on edaphoclimatic conditions such as light exposure, altitude, ultraviolet radiation, humidity, and temperature [42]. Thus, the differences observed across the various studies may be attributed to these factors, among others.

### 2.2. Anti-Inflammatory Activity

Inflammation is a response triggered by infectious agents (like bacteria, viruses, or fungi) or by non-infectious conditions such as tissue injury, cell death, cancer, ischemia, and degeneration, and the use of plants and their derivatives as anti-inflammatory agents dates back to ancient times [43].

Regarding the biological properties of the extracts, their anti-inflammatory activity was evaluated by assessing their ability to inhibit protein denaturation. Although this method is not a direct assay, it is commonly used to estimate the anti-inflammatory potential of plant samples [44].

Based on the results presented in Table 2, the samples with the lowest IC_50_ values—and thus the highest anti-inflammatory activity—were the *Umbilicus rupestris* leaves extract (URL), followed by the extracts of *H. stoechas* (HR) and *U. rupestris* flowers (URF).

Among the wild mountain plants harvested in the Serra da Gardunha region, *U. rupestris*, particularly its leaves (URL), proved to be the most promising in terms of anti-inflammatory properties. A quantitative comparison with the reference drug acetylsalicylic acid (IC_50_ = 4.20 μg/mL) indicates that, although URL (IC_50_ = 38.82 μg/mL) exhibits relevant anti-inflammatory activity, its potency is approximately 9 times lower than that of aspirin; thus, URL should not be regarded as a direct substitute for nonsteroidal anti-inflammatory drugs (NSAIDs). However, considering its natural origin and potentially reduced side effects, URL could serve as a promising natural source of bioactive compounds with potential for further development as complementary or alternative anti-inflammatory agents, particularly in cases where long-term NSAID use poses risks.

In folk medicine, *U. rupestris* is used against inflammation and irritation and diseases of the skin [46,47,48,49]. Regarding the anti-inflammatory activity, Benhouda and Yahia (2015) studied in vivo on Wistar rats the anti-inflammatory effects of methanolic extract of *U. rupestris* leaves by using several methods [49]. The methanolic extract induced a significant anti-inflammatory effect after the subcutaneous injection of the carrageenan solution. The effect of this extract on paw oedema induced by serotonin and histamine also showed a significant anti-inflammatory activity and dose dependent. This study confirmed the anti-inflammatory properties of *U. rupestris* methanolic extract from the leaves, supporting its traditional medicinal use [49]. These results agree with the ones of the present work, in which the plant *U. rupestris* has anti-inflammatory potential. Also, the anti-inflammatory activity of the *H. stoechas* extract was previously demonstrated, as reported by using different extracts and isolated compounds of *H. stoechas* [50,51,52,53]. Indeed, different compounds present in the *U. rupestris* and *H. stoechas* extracts have been shown to have anti-inflammatory activity, such as caffeic acid, coumarin, and rosmarinic acid [54,55,56,57]. Although these compounds are present in the other extracts analysed in our study, they did not show notable anti-inflammatory effects. Furthermore, the presence of apigenin and quercetin 3-*O*-rutinoside in the *U. rupestris* extracts, both compounds with previously reported anti-inflammatory activity [58,59], but not in those of *H. stoechas*, may account for the superior anti-inflammatory activity observed in *U. rupestris*, especially in the leaf extract.

### 2.3. Antioxidant Activity

The evaluation of the antioxidant activity in plant extracts is essential for identifying natural bioactive compounds that may contribute to the prevention of oxidative stress-related diseases and serve as safer alternatives to synthetic antioxidants in pharmaceutical and food applications [60,61,62].

The antioxidant properties of the extracts were evaluated using two different methodologies. The DPPH (2,2-diphenyl-1-picrylhydrazyl) assay was employed to determine whether the extracts exhibit antioxidant activity with respect to free radical scavenging. Additionally, the β-carotene-bleaching assay was used to assess the ability of the extracts to inhibit lipid peroxidation. The use of multiple methodologies allows for the evaluation of whether plant extracts possess various mechanisms of action related to antioxidant activity [63].

The DPPH methodology and Scherer and Godoy’s (2009) classification [45] showed that plants exhibiting the best antioxidant activity related to free radical scavenging were *C. salviifolius*, *C. vulgare*, and *G. prostrata.* Despite the fact that they display very strong antioxidant activity, their AAI values remain below that of gallic acid. In the study by Rebaya et al. [64], the aqueous extract (IC_50_ = 2.18 µg/mL) and the ethanolic extract (IC_50_ = 3.52 µg/mL) from *C. salviifolius* leaves exhibited activity comparable to the standard gallic acid (IC_50_ = 2.24 µg/mL), whereas the aqueous and ethanolic flower extracts showed lower activity (IC_50_ = 8.40 and 11.79 µg/mL, respectively), thus indicating that samples of these plants may exhibit activity similar to or slightly different from the gallic acid control. In terms of lipid peroxidation inhibition, although all exhibited weak activity (IC_50_ significantly greater compared to the BHT control), the *U. rupestris* showed a slightly better result (Table 2). It is worth noting that the different parts of these plants also influence their antioxidant activity, with the aerial parts of *C. salviifolius* and the flowers of *U. rupestris* showing the highest antioxidant activity. Petrova et al. (2023) evaluated the total polyphenol and flavonoid contents and antioxidant activity of freeze-dried aqueous extracts from different anatomical parts (leaves, flowers, and stems) of in vitro cultivated and wild-growing *C. vulgare* plants, demonstrating how the composition can vary between different parts of the plant and subsequently affect the bioactivity [42]. Several authors showed that different extracts and fractions of *C. salviifolius* may have high antioxidant properties through the radical scavenging activity, with possible correlations between the polyphenol composition and antioxidant activity of the extracts, with the polar extracts being more active than the non-polar ones [39,40,65,66]. In turn, the results reported herein and by other authors suggest that *C. salviifolius* is a weaker inhibitor of lipid peroxidation [41]. Also, in this work, the scavenging activity of the *C. vulgare* was demonstrated as strong, as previously reported [35,67,68,69,70].

The hydroethanolic extracts and decoction of *U. rupestris* demonstrated the ability to scavenge free radicals, inhibit lipid peroxidation, and prevent oxidative damage [71], consistent with the findings of this study on lipid peroxidation inhibition.

Comparing the extracts with the highest antioxidant activity—*C. salviifolius*, *C. vulgare*, and *G. prostrata*—the compounds (+)-gallocatechin 3-*O*-gallate and apigenin-7-glucoside were found in the *C. salviifolius* extracts but not in those of *C. vulgare* and *G. prostrata*. Previous studies have demonstrated the antioxidant activity of these compounds [72,73], suggesting that they may play a role in the slightly better antioxidant potential observed for *C. salviifolius*.

### 2.4. Antimicrobial Activity

Given the global rise in antimicrobial resistance, the investigation of plant extracts as potential sources of novel antimicrobial agents is crucial for the development of safer, more sustainable alternatives or complements to conventional antibiotics. In such a way, plants constitute an underexploited reservoir of antimicrobial agents, with potential applications in medicine, agriculture, and food preservation [74,75,76,77,78].

In the assays conducted to evaluate antimicrobial activity, several Gram-positive and Gram-negative bacterial species, as well as two yeast species, were studied using two different methodologies: disc diffusion susceptibility testing and determination of the minimum inhibitory concentration (MIC) of the extracts.

The results of the disc diffusion assay (Table 3) showed that the extracts of *C. vulgare* (CV), *Coincya monensis* (CMS and CMF), *Rubia peregrina* (RP), and *U. rupestris* (URL and URF) presented no antimicrobial activity against the tested microorganisms using this methodology (Table S8). These findings are broadly consistent with the MIC values obtained (Table 4).

Among the evaluated extracts, the most promising antimicrobial activity was observed for the *H. stoechas* extract (HS), followed by the extracts of *C. salviifolius* (CSAP and CSS). These extracts exhibited greater activity against Gram-positive bacterial species, as indicated by lower MIC values for these organisms. The antimicrobial activity of *C. salviifolius* and *H. stoechas* extracts was higher to the MIC values obtained for the antibiotic tetracycline. Regarding the yeast species, the extract of the flowers of *U. rupestris* (URF), along with the extracts of *H. stoechas* (HS) and *C. salviifolius* (CSAP and CSS), showed higher antimicrobial activity. The MIC values of the antifungal amphotericin B were lower than those of the *C. salviifolius*, *H. stoechas* and *U. rupestris* extracts. Concerning the obtained minimum lethal concentrations (MLC) values, most samples exhibited a bacteriostatic effect, with only the CSS, CV, GP, and HS extracts showing a bactericidal activity, primarily against Gram-negative bacteria.

*H. stoechas* is the plant with the most promising results, exhibiting the largest inhibition zones and the lowest minimum inhibitory concentrations against various Gram-positive and Gram-negative bacteria, as well as yeasts. The obtained results for this extract are in accordance with the results previously reported, demonstrating that different extracts of *H. stoechas* exhibit antimicrobial activity against these microorganisms [79,80,81,82].

Regarding *C. salviifolius*, the work of Mastino and collaborators (2021) showed that the butanolic and ethyl acetate fractions of *C. salviifolius* extract demonstrated predominant antimicrobial activity against *S. aureus*, while the aqueous fraction exhibited activity against both *S. aureus* and *Candida* spp. The authors concluded that these findings indicate that the extraction and partitioning processes influenced both the biological activity and the chemical composition of the *Cistus* extracts [39]. In another study, the ethanolic extract from *Cistus salviifolius* leaves exhibited activity against *L. monocytogenes* [83]. Although the *C. salviifolius* ethanolic extracts studied by Mahmoudi and collaborators, showed higher MIC values, they still demonstrated a bacteriostatic effect [40]. A study on organic extracts of *Cistus salviifolius* identified 2-acetylbenzoic acid as comprising approximately 3.9% of the analysed sample and associated these extracts with antimicrobial activity against ESKAPE clinical isolates [84].

Caffeic acid is a naturally occurring hydroxycinnamic acid, that exhibits a broad spectrum of bioactive properties. This compound has potent antioxidant activity by scavenging free radicals and reducing oxidative stress; can inhibit pro-inflammatory enzymes and cytokines, showing anti-inflammatory activity; possesses antimicrobial activity against various pathogens; and has antiproliferative and pro-apoptotic effects in cancer cell lines, suggesting potential therapeutic applications in oncology [56,57,85,86]. This compound is present in the extracts obtained from the plants *C. salviifolius,* and *H. stoechas* such as also reported in several studies [41,71,81,87,88], suggesting a potential role in the bioactive properties observed. The proposed mechanisms of action for antimicrobial activity involve cell membrane damage, inhibition of DNA or protein synthesis, and induction of oxidative stress [56,86]. Additionally, kaempferol is also present in samples of *C. salviifolius* [89,90]. Kaempferol is a naturally occurring flavonol that exhibits a wide range of pharmacological effects, including antioxidant, anti-inflammatory, antimicrobial, anticancer, antidiabetic, neuroprotective, and cardioprotective properties [91,92,93,94]. Kaempferol can inhibit the DNA gyrase and DNA helicases in methicillin-resistant *Staphylococcus aureus* [95,96]. In the case of *H. stoechas*, 3,4-dicaffeoylquinic acid (3,4-DCQA), present in its composition, can also be associated with its activity as isomers of caffeoylquinic acids can exhibit antimicrobial and efflux pump inhibitory activity against Gram-positive pathogenic bacteria [97]. A previous study has reported that 3,4-DCQA, also known as isochlorogenic acid C, can disrupt bacterial membranes by increasing permeability and causing leakage of intracellular contents, ultimately leading to cell death [98]. This mechanism is particularly effective against Gram-positive bacteria due to their simpler cell wall structure, which lacks the outer membrane found in Gram-negative bacteria [99]. Therefore, the strong antibacterial activity of HS may be associated, at least in part, with the membrane-damaging effects of 3,4-DCQA. Additionally, 4-hydroxyalternariol 9-methyl ether, also present in its composition, has been reported to also possess antibacterial activity [100]. These compounds are not present in the *C. salviifolius* extracts, which may suggest that they contribute to the enhanced antimicrobial activity of *H. stoechas*. Considering these, the antimicrobial activity of the *C. salviifolius* and *H. stoechas* extracts may possibly be attributed to the presence of these compounds or synergism among them.

### 2.5. Biocompatibility

Ensuring the safety of plant extracts for human consumption or application is crucial [32]. Thus, to evaluate the cytotoxic profile of the extracts on human cells, the current investigation was carried out by using a cultured Normal Human Dermal Fibroblasts (NHDF) cell line.

**Figure 1:**
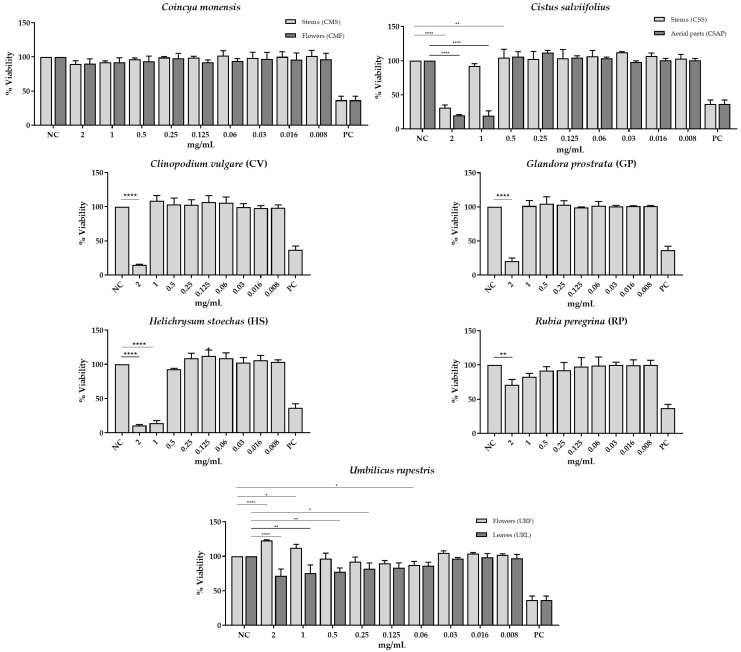
Biocompatibility of extracts for normal human dermal fibroblast (NHDF) cell line measured by MTT assay after 24 h of treatment. Negative control (NC) was performed using untreated cells and cells cultured with Fluorouracil (5-FU, 500 µg/mL) were used as positive control (PC). Results are expressed as means ± standard deviation of at least three independent experiments. The statistical analysis of the results was performed using the one-way ANOVA and Dunnett test, considering the values of *p* < 0.05 as statistically significant. * (*p* < 0.05); ** (*p* < 0.01); **** (*p* < 0.0001).

The results showed that the extract obtained from the aerial parts of *C. salviifolius* (CSAP) and *H. stoechas* (HS) were the most cytotoxic, reducing cell viability at lower concentrations (Figure 1). NHDF cell viability remained above 75% when exposed to extract concentrations of 1 mg/mL or lower, except for the extracts from the aerial parts of *C. salviifolius* (CSAP) and *H. stoechas* (HS) (IC_50_ < 1 mg/mL).

NHDF cells demonstrated high viability (higher than 70%) when incubated with the extracts of *C. monensis*, *R. peregrina*, and *U. rupestris*. The low cytotoxicity of *U. rupestris* extracts was supported in Human Umbilical Vein Endothelial Cells [101] and in non-tumour primary cell culture from porcine liver [71]. To the best of our knowledge, there are no studies evaluating the cytotoxicity of *C. monensis* and *R. peregrina*.

Despite the cytotoxicity of *C. salviifolius* aerial parts (CSAP) and *H. stoechas* (HS), they also demonstrated antimicrobial activity at concentrations below the IC_50_ found against NHDF cells, supporting their potential application as natural antimicrobial agents.

## 3. Materials and Methods

### 3.1. Collection of Plant Material

**Figure 2:**
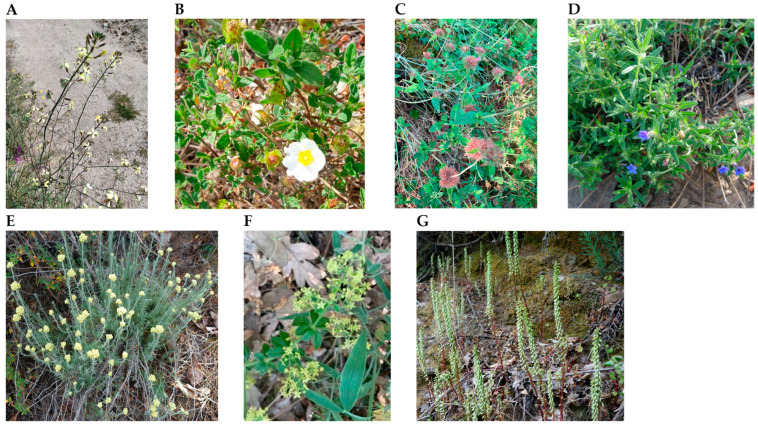
Photos of the plants collected in the Gardunha Mountain. Coincya monensis (**A**), Cistus salviifolius (**B**), Clinopodium vulgare (**C**), Glandora prostrata (**D**), Helichrysum stoechas (**E**), Rubia peregrina (**F**), Umbilicus rupestris (**G**).

The wild plants *Coincya monensis* (flowers, CMF or stems, CMS), *Cistus salviifolius* (aerial parts, CSAP or stems, CSS), *Clinopodium vulgare* (aerial parts, CV), *Glandora prostrata* (aerial parts, GP), *Helichrysum stoechas* (leaves and stems, HS), *Rubia peregrina* (aerial parts, RP), and *Umbilicus rupestris* (flowers, URF or leaves, URL) were collected in the northern area of Serra da Gardunha, Portugal, during spring of 2023 or spring of 2024 (Figure 2). The plant species were identified by technicians at the Biotech Plant Lab of Beira Interior, Castelo Branco, Portugal, and a voucher specimen was deposited in our laboratory. The plant materials were air-dried at room temperature, subsequently milled using a blade disintegrator and stored under the same conditions until further analysis.

### 3.2. Extraction

Milled plant material (10 g) was extracted with an ethanol/water 80:20 (200 mL) in an ultrasonic bath (Bransonic Ultrasonic Bath, Model M3800H-E, Brookfield, CT, USA) running continuously for 1 h, at room temperature. During extraction, the mixture was intermittently stirred with a spatula to unsure uniformity. At the end of each hour, the extract was removed, and an additional 200 mL of hydroethanolic solution was added. This process was repeated three times. The extracts were combined and centrifuged at 10,000× *g* for 20 min at 4 °C. The supernatant was removed and the ethanolic part evaporated under reduced pressure at 37 °C, and the aqueous part was freezing dried. The extracts were weighed, and the extraction yields were determined gravimetrically. The extracts were stored at −20 °C until use.

### 3.3. Phytochemical Analysis

#### 3.3.1. Determination of Total Phenolic Content

The total phenolic content of extracts was determined by the Folin-Ciocalteu colorimetric method according to Luís et al. [102]. Firstly, 450 µL of distilled water was added to 50 µL of each sample or gallic acid solution. Then, 2.5 mL of Folin-Ciocalteu’s reagent (Sigma-Aldrich, St. Louis, MO, USA) (0.2 N) was added and incubated for 5 min before the addition of 2 mL of aqueous Na_2_CO_3_ (LabKem, Barcelona, Spain) (75 g/L). The samples, diluted with methanol (VWR, Rosny-sous-Bois, France), were then incubated for 90 min at 30 °C. After incubation, the content of total phenolic compounds was determined by colorimetry at 765 nm. A calibration curve was prepared with methanolic solutions of gallic acid (purity 98%, TCI, Tokyo, Japan) in a range from 50 to 500 mg/L (*y =* 0.0012*x* + 0.0257, *R*^2^ = 0.99). The total phenolics were expressed in mg of gallic acid equivalents per gram of extract (mg GAE/g extract), and the analyses were performed in triplicate.

#### 3.3.2. Determination of Total Flavonoid Content

The total flavonoid content of extracts was determined by the aluminium chloride colorimetric method accordingly to Luís et al. [102]. A total of 1.5 mL of methanol, 0.1 mL of aluminium chloride (Scharlau, Barcelona, Spain, 10% *w*/*v*), 0.1 mL of 1 M potassium acetate (Fisher Chemical, Loughborough, UK) and 2.8 mL of distilled water were added to 500 µL of each extract diluted with methanol. Following a 30 min incubation at room temperature, the absorbance of the solutions was measured using a spectrophotometer (UV-6300PC, VWR, Lutterworth, Leicestershire, UK) at 415 nm. A calibration curve was made with methanolic solutions of quercetin (purity ≥ 95%, Sigma-Aldrich, USA) in a range from 12.5 to 200 mg/L (*y* = 0.0084*x* + 0.0006, *R*^2^ = 0.9993). The total flavonoids were expressed in mg quercetin equivalents per gram of extract (mg QE/g extract), and the analyses were performed in triplicate.

#### 3.3.3. Analysis of the Extracts Using Ultra-High-Performance Liquid Chromatography Coupled with Trapped Ion Mobility Spectrometry Time-of-Flight Mass Spectrometry (UHPLC timsTOF-MS)

To explore the phytochemical composition of the samples, a non-targeted metabolomics strategy was employed using ultra-high-performance liquid chromatography (UHPLC) coupled with trapped ion mobility time-of-flight mass spectrometry (timsTOF-MS; Bruker Daltonics, Bremen, Germany). The system was equipped with a VIP-HESI electrospray ionisation source to ensure efficient ion generation. Sample extracts were previously reconstituted in the minimum volume of the designated solvent system (ethanol/water 8:2), and 5 µL aliquots were injected into a ZORBAX Eclipse XDB-C18 rapid resolution HD column (2.1 × 100 mm, 1.8 µm; Agilent Technologies, Santa Clara, CA, USA).

Chromatographic separation was achieved using a binary solvent system composed of 0.1% formic acid in water (solvent A) and 0.1% formic acid in acetonitrile (solvent B). The gradient programme was as follows: the run began at 2% B (held for 1 min), increased gradually to 15% B by 7 min, and then ramped to 80% B over the next 8 min. Subsequently, the gradient rose to 100% B by 20 min and was maintained isocratically for 7 min. The system then returned to initial conditions, with a re-equilibration period of 2 min. The total runtime was 30 min, with a constant flow rate of 0.4 mL/min.

Mass spectrometric detection was performed in both positive and negative ionisation modes. Parameters included capillary voltages of ±4500 V and end plate offsets of ±500 V. Nitrogen was used as nebuliser gas (8 bar), drying gas (8 L/min at 240 °C), and sheath gas (4 L/min at 450 °C). Data acquisition ranged from *m*/*z* 20–1300, operating in both full-scan MS and MS/MS modes using Parallel Accumulation-Serial Fragmentation (PASEF). Ion mobility data were acquired within a 1/K_0_ range of 0.45–1.45 V·s/cm^2^ with a 100 ms ramp.

Raw data were processed using Bruker’s DataAnalysis (v6.1) and MetaboScape (v7.0.1). Feature extraction considered retention time (RT), *m*/*z*, and collisional cross section (CCS), with a signal intensity threshold of 10,000 counts. Tentative compound annotation was based on three strategies: spectral library matching (SL), comparison with an in-house analyte list (AL) of known phenolic compounds, and SmartFormula (SF) predictions constrained to CHNOPS atoms.

### 3.4. Anti-Inflammatory Activity

The in vitro anti-inflammatory activity was determined by evaluating the samples’ potential to inhibit protein denaturation according to a previous protocol [103]. Briefly, a solution of bovine serum albumin (BSA, 1% *w*/*v*, Sigma-Aldrich, USA) was prepared in phosphate-buffer solution (PBS, pH 6.8). The samples were diluted in dimethyl sulfoxide (DMSO), and 1 mL of each were preheated to 37 °C, and then, 9 mL of the BSA solution was added. The tubes were subsequently incubated for 10 min at 72 °C, followed by cooling on ice for another 10 min. Distilled water was used as a negative control and acetylsalicylic acid (purity > 99%, Sigma-Aldrich, USA) as a positive control. Finally, absorbance measurements were performed in triplicate using a spectrophotometer (Helios-Omega, Thermo Scientific, Waltham, MA, USA)) at 620 nm. The percentage of inhibition of protein denaturation was quantified using the equation below:

(1) $$\%\;Inhibition=100-\left(\left({Abs}_{sample}\times 100\right)/{Abs}_{control}\right)$$

where Abs_sample_ is the absorbance of each sample, and Abs_control_ is the absorbance of the control.

### 3.5. Antioxidant Activity

#### 3.5.1. DPPH Method

The free radical scavenging activity of the extracts was evaluated using the 2,2-diphenyl-1-picrylhydrazyl radical (DPPH) method as previously reported [102]. The crude extracts (25–250 mg/L) were compared with gallic acid (10, 25, 50, 75, 100 and 150 mg/L). A calibration curve was constructed with methanolic solutions of DPPH (Sigma-Aldrich, USA) in a range from 4.25 to 85 mg/L (*y* = 0.0084*x* + 0.0006, *R*^2^ = 0.999). Absorbances were measured at 517 nm, and the antioxidant activity was expressed through the Antioxidant Activity Index (AAI), calculated from the following equation:

(2) $$AAI=\left(final\;concentration\;of\;DPPH\;in\;the\;control\;sample\right)/{IC}_{50}$$

The antioxidant activity of the samples was classified as poor (AAI < 0.5), moderate (0.5 ≤ AAI < 1.0), strong (1.0 ≤ AAI < 2.0) or very strong (AAI ≥ 2.0) according to Scherer and Godoy [45]. The analyses were performed in triplicate.

#### 3.5.2. β-Carotene-Bleaching Assay

The ability of extracts to inhibit lipid peroxidation was evaluated by β-carotene bleaching assay [102,104]. For that, methanolic solutions of crude extracts were prepared with concentrations ranging 5 to 1000 mg/L. Butylated hydroxytoluene (BHT, purity 99%, Acros Organics, Geel, Belgium) was used as positive control using the same concentration range. The analyses were performed in duplicate. After acquisition of the absorbance values at 470 nm, the percentage of inhibition was calculated:

(3) $$\%\;Inhibition=\left(\left({Abs}_{sample}^{t=1h}-{Abs}_{control}^{t=1h}\right)/\left({Abs}_{control}^{t=0h}-{Abs}_{control}^{t=1h}\right)\right)\times 100$$

### 3.6. Antimicrobial Activity

#### 3.6.1. Plant Extracts, Microorganisms and Culture Media

The extracts were dissolved in dimethyl sulfoxide (DMSO, Fisher Chemical, UK) to a final concentration of 200 mg/mL and stored at −20 °C until use.

The Gram-positive bacteria used in this study included *Staphylococcus aureus* ATCC 25923 and the clinical isolate MRSA 12/08, *Bacillus cereus* ATCC 11778, and *Listeria monocytogenes* LMG 16779. The Gram-negative bacteria panel comprised *Escherichia coli* ATCC 25922, *Klebsiella pneumoniae* ATCC 13883, *Pseudomonas aeruginosa* ATCC 27853, *Salmonella* Typhimurium ATCC 13311, and *Acinetobacter baumannii* LMG 1025 and the clinical isolate AcB 13/10). In addition, two yeast species were tested: *Candida albicans* ATCC 90028 and *C. tropicalis* ATCC 750. The strains were obtained from American Type Culture Collection ATCC, USA) and the BCCM/LMG collection (Belgium), as indicated.

Müeller-Hinton agar (MHA, Biolife, Milan, Italy) and Müeller-Hinton broth (MHB, Biokar Diagnostics, Allonne, France) were used for growth and assays with the bacterial species, except for *L. monocytogenes*, for which Tryptone Soy Broth (TSB, VWR, Leuven, Belgium) and Tryptone Soy agar (TSA) were used. For yeasts, Sabouraud dextrose agar (SDA, Biokar Diagnostics, France) and Roswell Park Memorial Institute 1640 (RPMI 1640, Biochrom AG, Berlin, Germany) supplemented with 3-(N-morpholino)propanesulfonic acid (MOPS, TCI, Japan) were utilised.

All microorganisms used were cryopreserved at −80 °C in an appropriate cryoprotective medium containing 20% glycerol (Labchem, Santo Antão do Tojal, Portugal) for long-term storage. For short-term storage during experimental procedures, culture plates were maintained at 4 °C. The cultures were subcultured onto an appropriate solid medium and incubated at 37 °C for 24 h.

#### 3.6.2. Disc Diffusion Method

The disc diffusion method was performed to evaluate the susceptibility of the microorganisms to the extracts as described by Coimbra et al. [105]. Filter paper discs (6 mm) impregnated with 10 μL of each extract (2 mg/disc) or DMSO (100%, negative control) were placed on the surface of the inoculated plates. Inhibition zones were measured in millimetres, and the results are expressed as mean values ± standard deviations, based on a minimum of three independent assays.

#### 3.6.3. Determination of the Minimum Inhibitory Concentration (MIC)

The susceptibility of the microorganisms to the extracts was evaluated through the microdilution method accordingly to Coimbra et al. [104]. Briefly, the inoculum prepared by direct suspension in NaCl 0.85% (*w*/*v*) was adjusted to 0.5 McFarland and diluted in medium to obtain a final cell concentration of approximately 1 × 10^6^ colony-forming unit (CFU) per mL for bacteria and 1–5 × 10^3^ CFU/mL for yeasts. The assays were performed with a maximum concentration of 2 mg/mL of the extracts. Growth (negative) controls consisted of culture medium microbial suspension. A solvent control was prepared using DMSO at a maximum concentration of 1% (*v*/*v*) in presence of microbial suspension. A medium sterility control was included to confirm the absence of contamination. Positive controls with tetracycline (Sigma-Aldrich, USA) and amphotericin B (Sigma-Aldrich, USA) were also performed.

The 96-well plates were then incubated at 37 °C for 24 h for bacteria and 48 h for yeasts. Then, 30 μL of a 0.01% solution of resazurin (Sigma-Aldrich, USA) were added to the wells, followed by incubation for 2 h (bacteria) or 3 h (yeasts) at 37 °C. To establish minimum bactericidal concentrations (MLC), 10 µL of broth was removed from the well, inoculated on agar plates, and incubated at 37 °C for 24 h for bacteria and 48 h for yeasts. Inhibition of the test microorganism was evidenced by the unchanged blue colour of resazurin. The MLC was defined as the concentration required to kill 99.9% or more of the original microbial population. At least three independent determinations were performed, and the results were presented as modal values.

### 3.7. Evaluation of Extracts Biocompatibility

The cytotoxicity of the extracts was evaluated using Normal Human Dermal Fibroblasts (NHDF cells, ATCC PCS-201-012^TM^, Manassas, VA, USA), initially seeded in 96-well culture plates (100 µL) with 1 × 10^4^ cells/well in Roswell Park Memorial Institute (RPMI-1640, Gibco, Paisley, UK) supplemented with 10% FBS (PAN Biotech, Aidenbach, Germany) and 100 U/mL of penicillin G and 1 mg/mL of streptomycin, and grown with 5% CO_2_ at 37 °C for 24 h. Then, the culture medium was removed, and the cells were incubated with several concentrations of extracts for 24 h with RPMI supplemented with 10% FBS without antibiotics. Cells cultured with the well-established chemotherapy agent 5-fluorouracil (5-FU, 500 µg/mL, Sigma-Aldrich, USA) were used as a positive control, and those with only medium were used as a negative control. The 3-(4,5-dimethylthiazol-2-yl)-2,5-diphenyltetrazolium bromide (MTT, Sigma-Aldrich, USA) assay was used to evaluate cell metabolic function. For that, the medium was removed, and an RPMI solution with 0.5 mg/mL of MTT (100 µL in each well) was added to each sample. The plates were incubated in the same conditions for 3 h. Then, the pigmented formazan formed was dissolved with 100 µL of DMSO. Afterwards, the absorbance at 570 nm was read in a microplate spectrophotometer, Bio-Rad (Hercules, CA, USA) xMark. Cells at passages 26–29 and 35–41 were used for experiments. At least three independent determinations were performed.

### 3.8. Statistical Analysis

The statistical analysis of the results was performed using the one-way ANOVA and Dunnett test using the GraphPad Prism v8.01 software, with a 95% confidence interval, considering the values of *p* < 0.05 as statistically significant.

## 4. Conclusions

The chemical analysis of the studied wild mountain plants from the Serra da Gardunha region revealed that flavonoids and phenolic acids are the predominant bioactive compounds in their extracts. Among the plants evaluated, *Umbilicus rupestris*, particularly its leaves, emerged as the most promising candidate for anti-inflammatory applications, exhibiting a notable IC_50_ value of 38.82 ± 2.72 µg/mL. In terms of antioxidant potential, *Cistus salviifolius* demonstrated strong free radical scavenging activity, while *Umbilicus rupestris* achieved the highest lipid peroxidation inhibition. The evaluation of antimicrobial properties highlighted *Cistus salviifolius* and *Helichrysum stoechas* as the most effective species, showing larger inhibition zones and lower minimum inhibitory concentrations against a range of Gram-positive and Gram-negative bacteria, as well as yeasts. Notably, *H. stoechas* exhibited an exceptionally low MIC of 0.008 mg/mL against Gram-positive bacteria, underscoring its potent antimicrobial capacity.

Overall, the extracts from these wild mountain plants demonstrated significant bioactive potential across anti-inflammatory, antioxidant, and antimicrobial assays. These findings not only emphasise the value of *C. salviifolius* and *H. stoechas* as sources of novel bioactive compounds but also highlight the relevance of traditional medicinal plants in guiding pharmacological research. The study underscores the ecological and therapeutic importance of wild flora in the Serra da Gardunha and provides a strong foundation for future investigations into their chemical constituents and potential health applications.

## Figures and Tables

**Table 1:** Extraction yield and total phenolic and flavonoid contents in extracts. The results were expressed as mg of gallic acid equivalents per gram of extract (mg GAE/g extract) and mg of quercetin equivalents per gram of extract (mg QE/g extract), respectively, as mean ± standard deviation (n = 3).

Extracts	Extraction Yield (%)	Total Phenolic Content (mg GAE/g Extract)	Flavonoid Content (mg QE/g Extract)
CMF	38.95	6.60 ± 0.24	15.97 ± 0.79
CMS	7.68	12.88 ± 0.84	14.11 ± 0.7
CSAP	3.48	66.43 ± 1.45	31.69 ± 1.16
CSS	5.63	62.10 ± 2.96	15.16 ± 0.86
CV	6.23	64.77 ± 4.04	19.07 ± 0.61
GP	5.98	86.88 ± 3.47	18.50 ± 0.7
HS	4.28	57.88 ± 3.02	50.62 ± 1.51
RP	19.30	24.21 ± 1.17	15.53 ± 0.51
URF	16.50	48.43 ± 4.00	5.16 ± 0.68
URL	23.60	10.43 ± 1.15	6.57 ± 0.32

**Table 2:** Results of anti-inflammatory and antioxidant activity of extracts and standards (results expressed as mean ± standard deviation, n = 3). The statistical analysis of the results was performed using the one-way ANOVA and Dunnett test, considering the values of *p* < 0.05 as statistically significant.

	Anti-Inflammatory Activity * (µg/mL)	Antioxidant Activity			
		DPPH Method			β-Carotene Bleaching Assay
Samples		IC_50_ (µg/mL)	AAI *	Antioxidant Activity Classification	IC_50_ * (µg/mL)
CMF	371.93 ± 1.55	279.74 ± 57.57	0.21 ± 0.04	Poor	721.55 ± 9.03
CMS	402.71 ± 6.04	307.49 ± 57.57	0.20 ± 0.09	Poor	608.89 ± 9.39
CSAP	745.34 ± 31.96	19.18 ± 5.43	2.93 ± 0.12	Very strong	466.4 ± 10.71
CSS	700.42 ± 14.87	20.72 ± 5.73	2.84 ± 0.28	Very strong	342.82 ± 10.03
CV	309.25 ± 6.85	24.57 ± 7.52	2.32 ± 0.02	Very strong	696.49 ± 25.80
GP	276.31 ± 8.08	25.56 ± 7.80	2.21 ± 0.03	Very strong	352.72 ± 13.39
HS	51.75 ± 3.76	67.83 ± 18.87	0.83 ± 0.04	Moderate	433.09 ± 20.56
RP	304.11 ± 4.91	126.09 ± 78.59	0.55 ± 0.12	Moderate	621.47 ± 20.85
URF	54.79 ± 2.35	69.02 ± 25.72	0.81 ± 0.04	Moderate	335.57 ± 6.76
URL	38.82 ± 2.72	945.01 ± 393.37	0.29 ± 0.11	No activity	505.96 ± 16.77
Acetylsalicylic acid	4.20 ± 1.41	-	-	-	-
Gallic acid	-	3.92 ± 1.26	13.00 ± 0.67	Very strong	-
BHT	-	-	-	-	99.63 ± 10.76

* Statistical differences (*p* < 0.0001) were observed for all extracts in relation to the control, with the exception of the HS, URF and URL extracts in the anti-inflammatory assay, where no statistical difference was observed. AAI—Antioxidant activity index; BHT—Butylated Hydroxytoluene. The antioxidant activity of the samples was classified as: poor (AAI < 0.5), moderate (0.5 ≤ AAI < 1.0), strong (1.0 ≤ AAI < 2.0) or very strong (AAI ≥ 2.0) accordingly to Scherer and Godoy [45].

**Table 3:** Diameters of the inhibition halos (mm) in bacterial and yeast species are presented as mean ± standard deviation (at least three independent assays). Discs with a diameter of 6 mm were used.

	Inhibition Zone (10 µL/Disc)			
Species	CSAP	CSS	GP	HS
*Staphylococcus aureus* ATCC 25923	12.67 ± 0.78	11.72 ± 0.43	-	24.03 ± 2.21
*Staphylococcus aureus* MRSA 05/15	10.05 ± 0.78	10.77 ± 0.03	-	22.95 ± 0.92
*Bacillus cereus* ATCC 11778	8.52 ± 0.83	9.99 ± 0.42	-	21.01 ± 1.27
*Listeria monocytogenes* LMG 16779	10.55 ± 0.33	11.23 ± 0.57	-	28.91 ± 1.36
*Escherichia coli* ATCC 25922	-	-	-	-
*Klebsiella pneumoniae* ATCC 13883	8.39 ± 1.24	9.49 ± 0.43	8.63 ± 1.32	8.71 ± 2.18
*Pseudomonas aeruginosa* ATCC 27853	-	-	-	-
*Salmonella* Typhimurium ATCC 13311	-	-	-	-
*Acinetobacter baumannii* AcB 13/10	9.13 ± 0.11	7.67 ± 0.73	-	6.52 ± 0.75
*Acinetobacter baumannii* LMG 1025	9.11 ± 0.57	8.73 ± 0.53	-	6.3 ± 0.61
*Candida albicans* ATCC 90028	-	-	-	7.53 ± 2.64
*Candida tropicalis* ATCC 750	-	-	-	-

“-“ No activity

**Table 4:** Minimum inhibitory concentration (MIC, mg/mL) and minimum lethal concentration (MLC, mg/mL) of extracts on bacterial and yeast species presented as modal values.

	MIC (MLC)											
Species	CMF	CMS	CSAP	CSS	CV	GP	HS	RP	URF	URL	TET	AMP B
*Staphylococcus aureus* ATCC 25923	>2	>2	0.5 (2)	0.5 (2)	>2	2	0.008 (0.03)	>2	>2	>2	1	-
*Staphylococcus aureus* MRSA 05/15	>2	>2	0.25 (2)	0.5	>2	>2	0.008 (0.03)	>2	>2	>2	0.25	-
*Bacillus cereus* ATCC 11778	>2	>2	0.25	0.5	>2	>2	0.008 (0.25)	>2	>2	>2	0.06	-
*Listeria monocytogenes* LMG 16779	>2	>2	0.5 (2)	1	>2	>2	0.008 (0.125)	>2	>2	>2	0.25	-
*Escherichia coli* ATCC 25922	>2	>2	>2	>2	>2	>2	>2	>2	>2	>2	2	-
*Klebsiella pneumoniae* ATCC 13883	>2	>2	1	1	2 (2)	1 (1)	2 (2)	>2	>2	2	4	-
*Pseudomonas aeruginosa* ATCC 27853	>2	>2	>2	>2	>2	>2	>2	>2	>2	>2	16	-
*Salmonella* Typhimurium ATCC 13311	>2	>2	>2	>2	>2	>2	>2	>2	>2	>2	4	-
*Acinetobacter baumannii* AcB 13/10	>2	>2	2	2 (2)	>2	>2	2	>2	>2	>2	512	-
*Acinetobacter baumannii* LMG 1025	>2	>2	1	1 (1)	>2	>2	2 (2)	>2	>2	>2	4	-
*Candida albicans* ATCC 90028	>2	>2	0.03 (2)	0.016	>2	>2	0.5 (2)	>2	0.03	>2	-	0.5
*Candida tropicalis* ATCC 750	>2	>2	0.25 (0.5)	0.25 (1)	1 (1)	1 (2)	0.5 (1)	>2	0.25 (1)	>2	-	1

When no MLC value is reported, it indicates that the value is greater than 2 mg/mL. TET—tetracycline (μg/mL); AMP B—amphotericin B (μg/mL).

## Data Availability

Data is contained within the text.

## Supplementary Materials

The following supporting information can be downloaded at: https://www.mdpi.com/article/10.3390/molecules30193876/s1, Table S1. Representative compounds identified in *Cistus salviifolius* extracts using UHPLC-timsTOF-MS, based on combined annotation by spectral library matching, in-house analyte list, and SmartFormula. Identification supported by accurate mass, retention time, and collisional cross section. Table S2. Representative compounds identified in *Clinopodium vulgare* extract. Table S3. Representative compounds identified in *Coincya monensis* extracts. Table S4. Representative compounds identified in *Glandora prostrata* extract. Table S5. Representative compounds identified in *Helichrysum stoechas* extract. Table S6. Representative compounds identified in *Rubia peregrina* extract. Table S7. Representative compounds identified in *Umbilicus rupestris* extracts. Table S8. Diameters of the inhibition halos (mm) in bacterial and yeast species are presented as mean ± standard deviation (at least three independent assays). Discs with a diameter of 6 mm were used. Figure S1. Base peak chromatogram (BPC) in positive ion mode of the analysed samples. Figure S2. Base peak chromatogram (BPC) in negative ion mode of the analysed samples.
