# Supplementary material for: Bioactive Potential of Wild Plants from Gardunha Mountain: Phytochemical Characterization and Biological Activities
Source: Molecules. 2025 Sep 25;30(19):3876. doi: 10.3390/molecules30193876 (PMC12526268; doi:10.3390/molecules30193876)
Supplement: Supplementary file 1 [file molecules-30-03876-s001.zip › molecules-3817622-supplementary.pdf]

**Table S1.** Representative compounds identified in the *Cistus salviifolius* extracts using UHPLC–timsTOF–MS, based on combined annotation by spectral library matching, in-house analyte list, and SmartFormula. Identification supported by accurate mass, retention time, and collisional cross section.

| Compound Name | Formula | Measured *m/z* | Retention Time (min) | CCS (Å²) |
|---|---|---|---|---|
| | | ***Cistus salviifolius* aerial parts** | | |
| 2-Acetylbenzoic acid | $C_9H_8O_3$ | 165.05491 | 1.28 | 118.9 |
| Gallic acid | $C_7H_6O_5$ | 169.0144 | 1.5 | 121.1 |
| *p*-Coumaric acid 4-*O*-glucoside | $C_{15}H_{18}O_8$ | 325.09312 | 4.04 | 188.6 |
| Di-*O*-methylbergenin | $C_{16}H_{20}O_9$ | 355.10323 | 5.48 | 194.2 |
| 3-Feruloylquinic acid | $C_{17}H_{20}O_9$ | 367.10438 | 6.45 | 174.3 |
| Neochlorogenic acid | $C_{16}H_{18}O_9$ | 353.08781 | 6.59 | 180.3 |
| Caffeic acid | $C_9H_8O_4$ | 179.03469 | 6.73 | 128.4 |
| (+)-Gallocatechin 3-*O*-gallate | $C_{22}H_{18}O_{11}$ | 457.07672 | 7.87 | 198.3 |
| Kaempferol 3-*O*-glucosyl-rhamnosyl-galactoside | $C_{33}H_{40}O_{20}$ | 755.20215 | 8.49 | 250.0 |
| Coumarin | $C_9H_6O_2$ | 147.04417 | 8.55 | 113.9 |
| Myricetin 3-*O*-galactoside | $C_{21}H_{20}O_{13}$ | 479.08358 | 8.7 | 200.2 |
| Rhoifolin 4'-*O*-glucoside | $C_{33}H_{40}O_{19}$ | 739.20736 | 8.82 | 252.4 |
| Scopoletin | $C_{10}H_8O_4$ | 193.05015 | 9.04 | 123.2 |
| Quercetin 3-*O*-rutinoside | $C_{27}H_{30}O_{16}$ | 609.14555 | 9.08 | 227.1 |
| Myricetin 3-*O*-rhamnoside | $C_{21}H_{20}O_{12}$ | 463.08787 | 9.17 | 194.8 |
| Ellagic acid | $C_{14}H_6O_8$ | 300.99874 | 9.2 | 151.4 |
| Dihydroquercetin 3-*O*-rhamnoside | $C_{21}H_{22}O_{11}$ | 449.10984 | 9.21 | 185.3 |
| Apigenin 6,8-di-C-glucoside | $C_{27}H_{30}O_{15}$ | 593.15047 | 9.43 | 225.2 |
| Naringin | $C_{27}H_{32}O_{14}$ | 579.17105 | 9.5 | 214.1 |
| Quercetin 3-*O*-arabinoside | $C_{20}H_{18}O_{11}$ | 433.07686 | 9.58 | 194.0 |
| 6-Hydroxyluteolin 7-*O*-rhamnoside | $C_{21}H_{20}O_{11}$ | 447.09275 | 9.62 | 196.6 |
| Genistin | $C_{21}H_{20}O_{10}$ | 431.09793 | 9.7 | 203.7 |
| Apigenin-7-glucoside | $C_{21}H_{20}O_{10}$ | 431.09858 | 9.78 | 215.4 |
| 2'-Hydroxy-5'-methoxyacetophenone | $C_9H_{10}O_3$ | 165.05526 | 9.94 | 132.6 |
| Luteolin | $C_{15}H_{10}O_6$ | 287.05723 | 10.79 | 150.2 |
| 6-Hydroxyluteolin | $C_{15}H_{10}O_7$ | 301.03525 | 10.83 | 160.0 |

| | | | | |
|---|---|---|---|---|
| 7,3',4'-Trihydroxyflavone | $C_{15}H_{10}O_5$ | 269.04503 | 11.38 | 154.3 |
| Demethoxy-7-*O*-methylcapillarisin | $C_{16}H_{12}O_6$ | 299.05573 | 11.51 | 163.9 |
| Pterocarpadiol A | $C_{16}H_{12}O_7$ | 315.05089 | 11.62 | 167.2 |
| Hispidulin | $C_{16}H_{12}O_6$ | 299.05582 | 12.17 | 163.5 |
| Olivetol | $C_{11}H_{16}O_2$ | 181.12295 | 12.29 | 116.8 |
| Usnic acid | $C_{18}H_{16}O_7$ | 343.08199 | 12.7 | 180.5 |
| Rosmarinic acid | $C_{18}H_{16}O_8$ | 359.07671 | 12.74 | 178.5 |
| Glycitein | $C_{16}H_{12}O_5$ | 283.06084 | 12.87 | 163.9 |
| 6alpha-Hydroxymedicarpin | $C_{16}H_{14}O_5$ | 285.07641 | 12.91 | 165.9 |
| ***Cistus salviifolius* stems** | | | | |
| 2-Acetylbenzoic acid | $C_9H_8O_3$ | 165.05491 | 1.28 | 118.9 |
| Gallic acid | $C_7H_6O_5$ | 169.0144 | 1.5 | 121.1 |
| *p*-Coumaric acid 4-*O*-glucoside | $C_{15}H_{18}O_8$ | 325.09252 | 4.92 | 188.6 |
| (+)-Gallocatechin | $C_{15}H_{14}O_7$ | 305.06561 | 5.91 | 167.4 |
| Neochlorogenic acid | $C_{16}H_{18}O_9$ | 353.08781 | 6.59 | 180.3 |
| Caffeic acid | $C_9H_8O_4$ | 179.03469 | 6.73 | 128.4 |
| (+)-Gallocatechin 3-*O*-gallate | $C_{22}H_{18}O_{11}$ | 457.07672 | 7.87 | 198.3 |
| Kaempferol 3-*O*-glucosyl-rhamnosyl-galactoside | $C_{33}H_{40}O_{20}$ | 755.20215 | 8.49 | 250.0 |
| Coumarin | $C_9H_6O_2$ | 147.04417 | 8.55 | 113.9 |
| Myricetin 3-*O*-galactoside | $C_{21}H_{20}O_{13}$ | 479.08358 | 8.7 | 200.2 |
| Scopoletin | $C_{10}H_8O_4$ | 193.05015 | 9.04 | 123.2 |
| Quercetin 3-*O*-rutinoside | $C_{27}H_{30}O_{16}$ | 609.14555 | 9.08 | 227.1 |
| Myricetin 3-*O*-rhamnoside | $C_{21}H_{20}O_{12}$ | 463.08787 | 9.17 | 194.8 |
| Ellagic acid | $C_{14}H_6O_8$ | 300.99874 | 9.2 | 151.4 |
| Apigenin 6,8-di-C-glucoside | $C_{27}H_{30}O_{15}$ | 593.15047 | 9.43 | 225.2 |
| Naringin | $C_{27}H_{32}O_{14}$ | 579.17105 | 9.5 | 214.1 |
| Quercetin 3-*O*-arabinoside | $C_{20}H_{18}O_{11}$ | 433.07686 | 9.58 | 194.0 |
| 6-Hydroxyluteolin 7-*O*-rhamnoside | $C_{21}H_{20}O_{11}$ | 447.09275 | 9.62 | 196.6 |
| Genistin | $C_{21}H_{20}O_{10}$ | 431.09793 | 9.7 | 203.7 |
| Apigenin-7-glucoside | $C_{21}H_{20}O_{10}$ | 431.09858 | 9.78 | 215.4 |
| 2'-Hydroxy-5'-methoxyacetophenone | $C_9H_{10}O_3$ | 165.05526 | 9.94 | 132.6 |

| | | | | |
|---|---|---|---|---|
| Luteolin | $C_{15}H_{10}O_6$ | 287.05723 | 10.79 | 150.2 |
| 6-Hydroxyluteolin | $C_{15}H_{10}O_7$ | 301.03525 | 10.83 | 160.0 |
| 7,3',4'-Trihydroxyflavone | $C_{15}H_{10}O_5$ | 269.04503 | 11.38 | 154.3 |
| Demethoxy-7-*O*-methylcapillarisin | $C_{16}H_{12}O_6$ | 299.05573 | 11.51 | 163.9 |
| Pterocarpadiol A | $C_{16}H_{12}O_7$ | 315.05089 | 11.62 | 167.2 |
| Hispidulin | $C_{16}H_{12}O_6$ | 299.05582 | 12.17 | 163.5 |
| Usnic acid | $C_{18}H_{16}O_7$ | 343.08199 | 12.7 | 180.5 |
| Rosmarinic acid | $C_{18}H_{16}O_8$ | 359.07671 | 12.74 | 178.5 |
| Glycitein | $C_{16}H_{12}O_5$ | 283.06084 | 12.87 | 163.9 |

**Table S2.** Representative compounds identified in *Clinopodium vulgare* extract using UHPLC–timsTOF–MS, based on combined annotation by spectral library matching, in-house analyte list, and SmartFormula. Identification supported by accurate mass, retention time, and collisional cross section.

| Compound Name | Formula | Measured m/z | Retention Time (min) | CCS (Å²) |
|---|---|---|---|---|
| 2-Acetylbenzoic acid | $C_9H_8O_3$ | 165.05491 | 1.28 | 118.9 |
| Gallic acid | $C_7H_6O_5$ | 169.0144 | 1.5 | 121.1 |
| Neochlorogenic acid | $C_{16}H_{18}O_9$ | 353.08747 | 4.59 | 166.7 |
| Esculin | $C_{15}H_{16}O_9$ | 339.07098 | 4.68 | 170.4 |
| *p*-Coumaric acid 4-*O*-glucoside | $C_{15}H_{18}O_8$ | 325.09252 | 4.92 | 188.6 |
| 3-Feruloylquinic acid | $C_{17}H_{20}O_9$ | 367.10438 | 6.45 | 174.3 |
| Caffeic acid | $C_9H_8O_4$ | 179.03469 | 6.73 | 128.4 |
| Coumarin | $C_9H_6O_2$ | 147.04417 | 8.55 | 113.9 |
| Myricetin 3-*O*-galactoside | $C_{21}H_{20}O_{13}$ | 479.08358 | 8.7 | 200.2 |
| Scopoletin | $C_{10}H_8O_4$ | 193.05015 | 9.04 | 123.2 |
| Eriocitrin | $C_{27}H_{32}O_{15}$ | 595.16679 | 9.04 | 223.3 |
| 4-Hydroxyalternariol 9-methyl ether | $C_{15}H_{12}O_6$ | 289.07259 | 9.06 | 153.9 |
| Quercetin 3-*O*-rutinoside | $C_{27}H_{30}O_{16}$ | 609.14555 | 9.08 | 227.1 |
| Apigenin 6,8-di-C-glucoside | $C_{27}H_{30}O_{15}$ | 593.15048 | 9.1 | 227.4 |
| Ellagic acid | $C_{14}H_6O_8$ | 300.99874 | 9.2 | 151.4 |
| Dihydroquercetin 3-*O*-rhamnoside | $C_{21}H_{22}O_{11}$ | 449.10984 | 9.21 | 185.3 |
| Myricetin 3-*O*-rhamnoside | $C_{21}H_{20}O_{12}$ | 463.08804 | 9.27 | 197.6 |
| Naringin | $C_{27}H_{32}O_{14}$ | 579.17105 | 9.5 | 214.1 |
| 6-Hydroxyluteolin 7-*O*-rhamnoside | $C_{21}H_{20}O_{11}$ | 447.09275 | 9.62 | 196.6 |

| | | | | |
|---|---|---|---|---|
| Genistin | $C_{21}H_{20}O_{10}$ | 431.09793 | 9.7 | 203.7 |
| Luteolin | $C_{15}H_{10}O_6$ | 287.05723 | 10.79 | 150.2 |
| 6-Hydroxyluteolin | $C_{15}H_{10}O_7$ | 301.03525 | 10.83 | 160.0 |
| 7,3',4'-Trihydroxyflavone | $C_{15}H_{10}O_5$ | 269.04503 | 11.38 | 154.3 |
| Umbelliferone | $C_9H_6O_3$ | 161.02406 | 11.49 | 174.7 |
| Pterocarpadiol A | $C_{16}H_{12}O_7$ | 315.05089 | 11.62 | 167.2 |
| Demethoxy-7-*O*-methylcapillarisin | $C_{16}H_{12}O_6$ | 299.05561 | 11.73 | 163.9 |
| Hispidulin | $C_{16}H_{12}O_6$ | 299.05582 | 12.17 | 163.5 |
| Olivetol | $C_{11}H_{16}O_2$ | 181.12295 | 12.29 | 116.8 |
| Rosmarinic acid | $C_{18}H_{16}O_8$ | 359.07671 | 12.74 | 178.5 |
| Glycitein | $C_{16}H_{12}O_5$ | 283.06084 | 12.87 | 163.9 |
| 6alpha-Hydroxymedicarpin | $C_{16}H_{14}O_5$ | 285.07641 | 12.91 | 165.9 |
| Usnic acid | $C_{18}H_{16}O_7$ | 343.0823 | 16.25 | 172.5 |

**Table S3.** Representative compounds identified in *Coincya monensis* extracts using UHPLC–timsTOF–MS, based on combined annotation by spectral library matching, in-house analyte list, and SmartFormula. Identification supported by accurate mass, retention time, and collisional cross section.

| Compound Name | Formula | Measured *m/z* | Retention Time (min) | CCS (Å$^2$) |
|---|---|---|---|---|
| | | *Coincya monensis* Flowers | | |
| 2-Acetylbenzoic acid | $C_9H_8O_3$ | 165.05491 | 1.28 | 118.9 |
| Gallic acid | $C_7H_6O_5$ | 169.0144 | 1.5 | 121.1 |
| *p*-Coumaric acid 4-*O*-glucoside | $C_{15}H_{18}O_8$ | 325.09312 | 4.04 | 188.6 |
| Neochlorogenic acid | $C_{16}H_{18}O_9$ | 353.08787 | 6.04 | 146.0 |
| Caffeic acid | $C_9H_8O_4$ | 179.03469 | 6.73 | 128.4 |
| Coumarin | $C_9H_6O_2$ | 147.04417 | 8.55 | 113.9 |
| Scopoletin | $C_{10}H_8O_4$ | 193.05015 | 9.04 | 123.2 |
| Apigenin 6,8-di-C-glucoside | $C_{27}H_{30}O_{15}$ | 593.15048 | 9.1 | 227.4 |
| Myricetin 3-*O*-rhamnoside | $C_{21}H_{20}O_{12}$ | 463.08804 | 9.27 | 197.6 |
| Quercetin 3-*O*-arabinoside | $C_{20}H_{18}O_{11}$ | 433.07686 | 9.58 | 194.0 |
| 4-hydroxy-5-(3,4-dihydroxyphenyl)-valeric acid | $C_{11}H_{14}O_5$ | 227.09245 | 9.59 | 134.2 |
| 6-Hydroxyluteolin 7-*O*-rhamnoside | $C_{21}H_{20}O_{11}$ | 447.09275 | 9.62 | 196.6 |
| Luteolin | $C_{15}H_{10}O_6$ | 287.05723 | 10.79 | 150.2 |
| 6-Hydroxyluteolin | $C_{15}H_{10}O_7$ | 301.03525 | 10.83 | 160.0 |

| | | | | |
|---|---|---|---|---|
| 7,3',4'-Trihydroxyflavone | $C_{15}H_{10}O_5$ | 269.04503 | 11.38 | 154.3 |
| Pterocarpadiol A | $C_{16}H_{12}O_7$ | 315.05089 | 11.62 | 167.2 |
| Demethoxy-7-*O*-methylcapillarisin | $C_{16}H_{12}O_6$ | 299.05561 | 11.73 | 163.9 |
| Usnic acid | $C_{18}H_{16}O_7$ | 343.0823 | 16.25 | 172.5 |
| ***Coincya monensis* Stems** | | | | |
| 2-Acetylbenzoic acid | $C_9H_8O_3$ | 165.05491 | 1.28 | 118.9 |
| Gallic acid | $C_7H_6O_5$ | 169.0144 | 1.5 | 121.1 |
| *p*-Coumaric acid 4-*O*-glucoside | $C_{15}H_{18}O_8$ | 325.09252 | 4.92 | 188.6 |
| Neochlorogenic acid | $C_{16}H_{18}O_9$ | 353.08787 | 6.04 | 146.0 |
| 3-Feruloylquinic acid | $C_{17}H_{20}O_9$ | 367.10438 | 6.45 | 174.3 |
| Caffeic acid | $C_9H_8O_4$ | 179.03469 | 6.73 | 128.4 |
| Coumarin | $C_9H_6O_2$ | 147.04417 | 8.55 | 113.9 |
| Scopoletin | $C_{10}H_8O_4$ | 193.05015 | 9.04 | 123.2 |
| Apigenin 6,8-di-C-glucoside | $C_{27}H_{30}O_{15}$ | 593.15048 | 9.1 | 227.4 |
| Ellagic acid | $C_{14}H_6O_8$ | 300.99874 | 9.2 | 151.4 |
| Dihydroquercetin 3-*O*-rhamnoside | $C_{21}H_{22}O_{11}$ | 449.10984 | 9.21 | 185.3 |
| Myricetin 3-*O*-rhamnoside | $C_{21}H_{20}O_{12}$ | 463.08804 | 9.27 | 197.6 |
| Naringin | $C_{27}H_{32}O_{14}$ | 579.17105 | 9.5 | 214.1 |
| Quercetin 3-*O*-arabinoside | $C_{20}H_{18}O_{11}$ | 433.07686 | 9.58 | 194.0 |
| 4-hydroxy-5-(3,4-dihydroxyphenyl)-valeric acid | $C_{11}H_{14}O_5$ | 227.09245 | 9.59 | 134.2 |
| 6-Hydroxyluteolin 7-*O*-rhamnoside | $C_{21}H_{20}O_{11}$ | 447.09275 | 9.62 | 196.6 |
| 6-Hydroxyluteolin | $C_{15}H_{10}O_7$ | 301.03525 | 10.83 | 160.0 |
| Pterocarpadiol A | $C_{16}H_{12}O_7$ | 315.05089 | 11.62 | 167.2 |
| Demethoxy-7-*O*-methylcapillarisin | $C_{16}H_{12}O_6$ | 299.05561 | 11.73 | 163.9 |
| Olivetol | $C_{11}H_{16}O_2$ | 181.12295 | 12.29 | 116.8 |
| Usnic acid | $C_{18}H_{16}O_7$ | 343.08199 | 12.7 | 180.5 |

**Table S4.** Representative compounds identified in *Glandora prostrata* extract using UHPLC–timsTOF–MS, based on combined annotation by spectral library matching, in-house analyte list, and SmartFormula. Identification supported by accurate mass, retention time, and collisional cross section.

| Compound Name | Formula | Measured *m/z* | Retention Time (min) | CCS (Å²) |
|---|---|---|---|---|
| 2-Acetylbenzoic acid | $C_9H_8O_3$ | 165.05491 | 1.28 | 118.9 |
| Gallic acid | $C_7H_6O_5$ | 169.0144 | 1.5 | 121.1 |
| *p*-Coumaric acid 4-*O*-glucoside | $C_{15}H_{18}O_8$ | 325.09312 | 4.04 | 188.6 |
| Neochlorogenic acid | $C_{16}H_{18}O_9$ | 353.08758 | 4.05 | 240.5 |
| Caffeic acid | $C_9H_8O_4$ | 179.03469 | 6.73 | 128.4 |
| Kaempferol 3-*O*-glucosyl-rhamnosyl-galactoside | $C_{33}H_{40}O_{20}$ | 755.20215 | 8.49 | 250.0 |
| Coumarin | $C_9H_6O_2$ | 147.04417 | 8.55 | 113.9 |
| Rhoifolin 4'-*O*-glucoside | $C_{33}H_{40}O_{19}$ | 739.20736 | 8.82 | 252.4 |
| Scopoletin | $C_{10}H_8O_4$ | 193.05015 | 9.04 | 123.2 |
| Quercetin 3-*O*-rutinoside | $C_{27}H_{30}O_{16}$ | 609.14555 | 9.08 | 227.1 |
| Apigenin 6,8-di-C-glucoside | $C_{27}H_{30}O_{15}$ | 593.15048 | 9.1 | 227.4 |
| Dihydroquercetin 3-*O*-rhamnoside | $C_{21}H_{22}O_{11}$ | 449.10984 | 9.21 | 185.3 |
| Myricetin 3-*O*-rhamnoside | $C_{21}H_{20}O_{12}$ | 463.08804 | 9.27 | 197.6 |
| Naringin | $C_{27}H_{32}O_{14}$ | 579.17105 | 9.5 | 214.1 |
| 4-hydroxy-5-(3,4-dihydroxyphenyl)-valeric acid | $C_{11}H_{14}O_5$ | 227.09245 | 9.59 | 134.2 |
| 6-Hydroxyluteolin 7-*O*-rhamnoside | $C_{21}H_{20}O_{11}$ | 447.09275 | 9.62 | 196.6 |
| Genistin | $C_{21}H_{20}O_{10}$ | 431.09793 | 9.7 | 203.7 |
| Luteolin | $C_{15}H_{10}O_6$ | 287.05723 | 10.79 | 150.2 |
| 6-Hydroxyluteolin | $C_{15}H_{10}O_7$ | 301.03525 | 10.83 | 160.0 |
| 7,3',4'-Trihydroxyflavone | $C_{15}H_{10}O_5$ | 269.04503 | 11.38 | 154.3 |
| Umbelliferone | $C_9H_6O_3$ | 161.02406 | 11.49 | 174.7 |
| Demethoxy-7-*O*-methylcapillarisin | $C_{16}H_{12}O_6$ | 299.05573 | 11.51 | 163.9 |
| Pterocarpadiol A | $C_{16}H_{12}O_7$ | 315.05089 | 11.62 | 167.2 |
| Hispidulin | $C_{16}H_{12}O_6$ | 299.05582 | 12.17 | 163.5 |
| Olivetol | $C_{11}H_{16}O_2$ | 181.12295 | 12.29 | 116.8 |
| Usnic acid | $C_{18}H_{16}O_7$ | 343.08199 | 12.7 | 180.5 |
| Rosmarinic acid | $C_{18}H_{16}O_8$ | 359.07671 | 12.74 | 178.5 |
| Glycitein | $C_{16}H_{12}O_5$ | 283.06084 | 12.87 | 163.9 |

**Table S5.** Representative compounds identified in the *Helichrysum stoechas* extract using UHPLC–timsTOF–MS, based on combined annotation by spectral library matching, in-house analyte list, and SmartFormula. Identification supported by accurate mass, retention time, and collisional cross section.

| Compound Name | Formula | Measured *m/z* | Retention Time (min) | CCS (Å²) |
|---|---|---|---|---|
| 2-Acetylbenzoic acid | $C_9H_8O_3$ | 165.05491 | 1.28 | 118.9 |
| Gallic acid | $C_7H_6O_5$ | 169.0144 | 1.5 | 121.1 |
| *p*-Coumaric acid 4-*O*-glucoside | $C_{15}H_{18}O_8$ | 325.09312 | 4.04 | 188.6 |
| Neochlorogenic acid | $C_{16}H_{18}O_9$ | 353.08758 | 4.05 | 240.5 |
| 3-Feruloylquinic acid | $C_{17}H_{20}O_9$ | 367.10438 | 6.45 | 174.3 |
| Caffeic acid | $C_9H_8O_4$ | 179.03469 | 6.73 | 128.4 |
| 3,4-Dicaffeoylquinic acid | $C_{25}H_{24}O_{12}$ | 515.11828 | 8.03 | 208.3 |
| Coumarin | $C_9H_6O_2$ | 147.04417 | 8.55 | 113.9 |
| Myricetin 3-*O*-rhamnoside | $C_{21}H_{20}O_{12}$ | 463.08711 | 8.68 | 198.8 |
| Myricetin 3-*O*-galactoside | $C_{21}H_{20}O_{13}$ | 479.08358 | 8.7 | 200.2 |
| 2,3-Dihydroxy-1-guaiacylpropanone | $C_{10}H_{12}O_5$ | 213.07637 | 8.97 | 130.1 |
| Scopoletin | $C_{10}H_8O_4$ | 193.05015 | 9.04 | 123.2 |
| 4-Hydroxyalternariol 9-methyl ether | $C_{15}H_{12}O_6$ | 289.07259 | 9.06 | 153.9 |
| Ellagic acid | $C_{14}H_6O_8$ | 300.99874 | 9.2 | 151.4 |
| Dihydroquercetin 3-*O*-rhamnoside | $C_{21}H_{22}O_{11}$ | 449.10984 | 9.21 | 185.3 |
| 4-hydroxy-5-(3,4-dihydroxyphenyl)-valeric acid | $C_{11}H_{14}O_5$ | 227.09245 | 9.59 | 134.2 |
| 6-Hydroxyluteolin 7-*O*-rhamnoside | $C_{21}H_{20}O_{11}$ | 447.09275 | 9.62 | 196.6 |
| Genistin | $C_{21}H_{20}O_{10}$ | 431.09793 | 9.7 | 203.7 |
| Luteolin | $C_{15}H_{10}O_6$ | 287.05723 | 10.79 | 150.2 |
| 6-Hydroxyluteolin | $C_{15}H_{10}O_7$ | 301.03525 | 10.83 | 160.0 |
| 7,3',4'-Trihydroxyflavone | $C_{15}H_{10}O_5$ | 269.04503 | 11.38 | 154.3 |
| Demethoxy-7-*O*-methylcapillarisin | $C_{16}H_{12}O_6$ | 299.05573 | 11.51 | 163.9 |
| Pterocarpadiol A | $C_{16}H_{12}O_7$ | 315.05089 | 11.62 | 167.2 |
| 2-Hydroxy-3,4-dimethoxybenzoic acid | $C_9H_{10}O_5$ | 197.04479 | 12.27 | 188.8 |
| Olivetol | $C_{11}H_{16}O_2$ | 181.12295 | 12.29 | 116.8 |
| Usnic acid | $C_{18}H_{16}O_7$ | 343.08199 | 12.7 | 180.5 |
| Rosmarinic acid | $C_{18}H_{16}O_8$ | 359.07671 | 12.74 | 178.5 |
| Dimethyl phthalate | $C_{10}H_{10}O_4$ | 195.06566 | 16.14 | 121.4 |

**Table S6.** Representative compounds identified in *Rubia peregrina* extract using UHPLC–timsTOF–MS, based on combined annotation by spectral library matching, in-house analyte list, and SmartFormula. Identification supported by accurate mass, retention time, and collisional cross section.

| Compound Name | Formula | Measured *m*/*z* | Retention Time (min) | CCS (Å²) |
|---|---|---|---|---|
| 2-Acetylbenzoic acid | $C_9H_8O_3$ | 165.05491 | 1.28 | 118.9 |
| Gallic acid | $C_7H_6O_5$ | 169.0144 | 1.5 | 121.1 |
| Neochlorogenic acid | $C_{16}H_{18}O_9$ | 353.08758 | 4.05 | 240.5 |
| *p*-Coumaric acid 4-*O*-glucoside | $C_{15}H_{18}O_8$ | 325.0927 | 4.32 | 188.6 |
| Caffeic acid | $C_9H_8O_4$ | 179.03434 | 4.35 | 181.5 |
| Esculin | $C_{15}H_{16}O_9$ | 339.07098 | 4.68 | 170.4 |
| Di-*O*-methylbergenin | $C_{16}H_{20}O_9$ | 355.10323 | 5.48 | 194.2 |
| 3-Feruloylquinic acid | $C_{17}H_{20}O_9$ | 367.10438 | 6.45 | 174.3 |
| Scopoletin | $C_{10}H_8O_4$ | 193.05043 | 6.53 | 115.9 |
| Kaempferol 3-*O*-glucosyl-rhamnosyl-galactoside | $C_{33}H_{40}O_{20}$ | 755.20215 | 8.49 | 250.0 |
| Coumarin | $C_9H_6O_2$ | 147.04417 | 8.55 | 113.9 |
| Myricetin 3-*O*-galactoside | $C_{21}H_{20}O_{13}$ | 479.08358 | 8.7 | 200.2 |
| Eriocitrin | $C_{27}H_{32}O_{15}$ | 595.16679 | 9.04 | 223.3 |
| Quercetin 3-*O*-rutinoside | $C_{27}H_{30}O_{16}$ | 609.14555 | 9.08 | 227.1 |
| Apigenin 6,8-di-C-glucoside | $C_{27}H_{30}O_{15}$ | 593.15048 | 9.1 | 227.4 |
| Ellagic acid | $C_{14}H_6O_8$ | 300.99874 | 9.2 | 151.4 |
| Myricetin 3-*O*-rhamnoside | $C_{21}H_{20}O_{12}$ | 463.08804 | 9.27 | 197.6 |
| Naringin | $C_{27}H_{32}O_{14}$ | 579.17105 | 9.5 | 214.1 |
| 3,4-Dicaffeoylquinic acid | $C_{25}H_{24}O_{12}$ | 515.11839 | 9.51 | 162.5 |
| Quercetin 3-*O*-arabinoside | $C_{20}H_{18}O_{11}$ | 433.07686 | 9.58 | 194.0 |
| 6-Hydroxyluteolin 7-*O*-rhamnoside | $C_{21}H_{20}O_{11}$ | 447.09275 | 9.62 | 196.6 |
| Genistin | $C_{21}H_{20}O_{10}$ | 431.09793 | 9.7 | 203.7 |
| Luteolin | $C_{15}H_{10}O_6$ | 287.05723 | 10.79 | 150.2 |
| 6-Hydroxyluteolin | $C_{15}H_{10}O_7$ | 301.03525 | 10.83 | 160.0 |
| 7,3',4'-Trihydroxyflavone | $C_{15}H_{10}O_5$ | 269.04503 | 11.38 | 154.3 |
| Umbelliferone | $C_9H_6O_3$ | 161.02406 | 11.49 | 174.7 |
| Demethoxy-7-*O*-methylcapillarisin | $C_{16}H_{12}O_6$ | 299.05573 | 11.51 | 163.9 |
| Pterocarpadiol A | $C_{16}H_{12}O_7$ | 315.05089 | 11.62 | 167.2 |
| Hispidulin | $C_{16}H_{12}O_6$ | 299.05582 | 12.17 | 163.5 |
| Olivetol | $C_{11}H_{16}O_2$ | 181.12295 | 12.29 | 116.8 |
| Usnic acid | $C_{18}H_{16}O_7$ | 343.08199 | 12.7 | 180.5 |

| | | | | |
|---|---|---|---|---|
| Rosmarinic acid | $C_{18}H_{16}O_8$ | 359.07671 | 12.74 | 178.5 |
| 6alpha-Hydroxymedicarpin | $C_{16}H_{14}O_5$ | 285.07641 | 12.91 | 165.9 |

**Table S7.** Representative compounds identified in *Umbilicus rupestris* extracts using UHPLC–timsTOF–MS, based on combined annotation by spectral library matching, in-house analyte list, and SmartFormula. Identification supported by accurate mass, retention time, and collisional cross section.

| **Compound Name** | **Formula** | **Measured *m*/*z*** | **Retention Time (min)** | **CCS (Å²)** |
|---|---|---|---|---|
| | | ***Umbilicus rupestris* Flowers** | | |
| 2-Acetylbenzoic acid | $C_9H_8O_3$ | 165.05491 | 1.28 | 118.9 |
| Gallic acid | $C_7H_6O_5$ | 169.0144 | 1.5 | 121.1 |
| *p*-Coumaric acid 4-*O*-glucoside | $C_{15}H_{18}O_8$ | 325.0927 | 4.32 | 188.6 |
| Caffeic acid | $C_9H_8O_4$ | 179.03434 | 4.35 | 181.5 |
| Esculin | $C_{15}H_{16}O_9$ | 339.07098 | 4.68 | 170.4 |
| Di-*O*-methylbergenin | $C_{16}H_{20}O_9$ | 355.10323 | 5.48 | 194.2 |
| (+)-Gallocatechin | $C_{15}H_{14}O_7$ | 305.06561 | 5.91 | 167.4 |
| Neochlorogenic acid | $C_{16}H_{18}O_9$ | 353.08787 | 6.04 | 146.0 |
| (+)-Gallocatechin 3-*O*-gallate | $C_{22}H_{18}O_{11}$ | 457.07672 | 7.87 | 198.3 |
| Coumarin | $C_9H_6O_2$ | 147.04417 | 8.55 | 113.9 |
| Myricetin 3-*O*-rhamnoside | $C_{21}H_{20}O_{12}$ | 463.08711 | 8.68 | 198.8 |
| Myricetin 3-*O*-galactoside | $C_{21}H_{20}O_{13}$ | 479.08358 | 8.7 | 200.2 |
| Scopoletin | $C_{10}H_8O_4$ | 193.05015 | 9.04 | 123.2 |
| 4-Hydroxyalternariol 9-methyl ether | $C_{15}H_{12}O_6$ | 289.07259 | 9.06 | 153.9 |
| Quercetin 3-*O*-rutinoside | $C_{27}H_{30}O_{16}$ | 609.14555 | 9.08 | 227.1 |
| Ellagic acid | $C_{14}H_6O_8$ | 300.99874 | 9.2 | 151.4 |
| Apigenin 6,8-di-C-glucoside | $C_{27}H_{30}O_{15}$ | 593.15047 | 9.43 | 225.2 |
| 6-Hydroxyluteolin 7-*O*-rhamnoside | $C_{21}H_{20}O_{11}$ | 447.09275 | 9.62 | 196.6 |
| 2'-Hydroxy-5'-methoxyacetophenone | $C_9H_{10}O_3$ | 165.05526 | 9.94 | 132.6 |
| Luteolin | $C_{15}H_{10}O_6$ | 287.05723 | 10.79 | 150.2 |
| 6-Hydroxyluteolin | $C_{15}H_{10}O_7$ | 301.03525 | 10.83 | 160.0 |
| 7,3',4'-Trihydroxyflavone | $C_{15}H_{10}O_5$ | 269.04503 | 11.38 | 154.3 |
| Demethoxy-7-*O*-methylcapillarisin | $C_{16}H_{12}O_6$ | 299.05573 | 11.51 | 163.9 |
| Pterocarpadiol A | $C_{16}H_{12}O_7$ | 315.05089 | 11.62 | 167.2 |
| Hispidulin | $C_{16}H_{12}O_6$ | 299.05582 | 12.17 | 163.5 |
| Olivetol | $C_{11}H_{16}O_2$ | 181.12295 | 12.29 | 116.8 |

| | | | | |
|---|---|---|---|---|
| Usnic acid | $C_{18}H_{16}O_7$ | 343.08199 | 12.7 | 180.5 |
| Rosmarinic acid | $C_{18}H_{16}O_8$ | 359.07671 | 12.74 | 178.5 |
| Glycitein | $C_{16}H_{12}O_5$ | 283.06084 | 12.87 | 163.9 |
| 6alpha-Hydroxymedicarpin | $C_{16}H_{14}O_5$ | 285.07641 | 12.91 | 165.9 |
| | | ***Umbilicus rupestris* Leaves** | | |
| 2-Acetylbenzoic acid | $C_9H_8O_3$ | 165.05491 | 1.28 | 118.9 |
| Gallic acid | $C_7H_6O_5$ | 169.0144 | 1.5 | 121.1 |
| *p*-Coumaric acid 4-*O*-glucoside | $C_{15}H_{18}O_8$ | 325.09312 | 4.04 | 188.6 |
| Caffeic acid | $C_9H_8O_4$ | 179.03434 | 4.35 | 181.5 |
| Neochlorogenic acid | $C_{16}H_{18}O_9$ | 353.08747 | 4.59 | 166.7 |
| (+)-Gallocatechin | $C_{15}H_{14}O_7$ | 305.06561 | 5.91 | 167.4 |
| (+)-Gallocatechin 3-*O*-gallate | $C_{22}H_{18}O_{11}$ | 457.07672 | 7.87 | 198.3 |
| Myricetin 3-*O*-rhamnoside | $C_{21}H_{20}O_{12}$ | 463.08711 | 8.68 | 198.8 |
| Myricetin 3-*O*-galactoside | $C_{21}H_{20}O_{13}$ | 479.08358 | 8.7 | 200.2 |
| 2,3-Dihydroxy-1-guaiacylpropanone | $C_{10}H_{12}O_5$ | 213.07637 | 8.97 | 130.1 |
| Quercetin 3-*O*-rutinoside | $C_{27}H_{30}O_{16}$ | 609.14555 | 9.08 | 227.1 |
| Ellagic acid | $C_{14}H_6O_8$ | 300.99874 | 9.2 | 151.4 |
| Sinapaldehyde | $C_{11}H_{12}O_4$ | 209.08154 | 9.42 | 126.8 |
| Apigenin 6,8-di-C-glucoside | $C_{27}H_{30}O_{15}$ | 593.15047 | 9.43 | 225.2 |
| Quercetin 3-*O*-arabinoside | $C_{20}H_{18}O_{11}$ | 433.07686 | 9.58 | 194.0 |
| 6-Hydroxyluteolin 7-*O*-rhamnoside | $C_{21}H_{20}O_{11}$ | 447.09275 | 9.62 | 196.6 |
| 2'-Hydroxy-5'-methoxyacetophenone | $C_9H_{10}O_3$ | 165.05526 | 9.94 | 132.6 |
| Luteolin | $C_{15}H_{10}O_6$ | 287.05723 | 10.79 | 150.2 |
| 6-Hydroxyluteolin | $C_{15}H_{10}O_7$ | 301.03525 | 10.83 | 160.0 |
| 7,3',4'-Trihydroxyflavone | $C_{15}H_{10}O_5$ | 269.04503 | 11.38 | 154.3 |
| Umbelliferone | $C_9H_6O_3$ | 161.02406 | 11.49 | 174.7 |
| Pterocarpadiol A | $C_{16}H_{12}O_7$ | 315.05089 | 11.62 | 167.2 |
| Demethoxy-7-*O*-methylcapillarisin | $C_{16}H_{12}O_6$ | 299.05561 | 11.73 | 163.9 |
| Hispidulin | $C_{16}H_{12}O_6$ | 299.05582 | 12.17 | 163.5 |
| Olivetol | $C_{11}H_{16}O_2$ | 181.12295 | 12.29 | 116.8 |
| Usnic acid | $C_{18}H_{16}O_7$ | 343.08199 | 12.7 | 180.5 |
| Rosmarinic acid | $C_{18}H_{16}O_8$ | 359.07671 | 12.74 | 178.5 |

**Table S8.** Diameters of the inhibition halos (mm) in bacterial and yeast species are presented as mean ± standard deviation (at least three independent assays). Discs with a diameter of 6 mm were used.

| | Inhibition Zone (10 μL/Disc) | | | | | | | | | |
|---|---|---|---|---|---|---|---|---|---|---|
| **Species** | **CMF** | **CMS** | **CSAP** | **CSS** | **CV** | **GP** | **HS** | **RP** | **URF** | **URL** |
| *Staphylococcus aureus* ATCC 25923 | - | - | 12.67 ± 0.78 | 11.72 ± 0.43 | - | - | 24.03 ± 2.21 | - | - | - |
| *Staphylococcus aureus* MRSA 05/15 | - | - | 10.05 ± 0.78 | 10.77 ± 0.03 | - | - | 22.95 ± 0.92 | - | - | - |
| *Bacillus cereus* ATCC 11778 | - | - | 8.52 ± 0.83 | 9.99 ± 0.42 | - | - | 21.01 ± 1.27 | - | - | - |
| *Listeria monocytogenes* LMG 16779 | - | - | 10.55 ± 0.33 | 11.23 ± 0.57 | - | - | 28.91 ± 1.36 | - | - | - |
| *Escherichia coli* ATCC 25922 | - | - | - | - | - | - | - | - | - | - |
| *Klebsiella pneumoniae* ATCC 13883 | - | - | 8.39 ± 1.24 | 9.49 ± 0.43 | - | 8.63 ± 1.32 | 8.71 ± 2.18 | - | - | - |
| *Pseudomonas aeruginosa* ATCC 27853 | - | - | - | - | - | - | - | - | - | - |
| *Salmonella* Typhimurium ATCC 13311 | - | - | - | - | - | - | - | - | - | - |
| *Acinetobacter baumannii* AcB 13/10 | - | - | 9.13 ± 0.11 | 7.67 ± 0.73 | - | - | 6.52 ± 0.75 | - | - | - |
| *Acinetobacter baumannii* LMG 1025 | - | - | 9.11 ± 0.57 | 8.73 ± 0.53 | - | - | 6.3 ± 0.61 | - | - | - |
| *Candida albicans* ATCC 90028 | - | - | - | - | - | - | 7.53 ± 2.64 | - | - | - |
| *Candida tropicalis* ATCC 750 | - | - | - | - | - | - | - | - | - | - |

CMF - *Coincya monensis* flowers; CMS - *Coincya monensis* stems; CSAP - *Cistus salviifolius* aerial parts; CSS - *Cistus salviifolius* stems; CV - *Clinopodium vulgare*; GP - *Glandora prostrata*; HS - *Helichrysum stoechas*; RP - *Rubia peregrina*; URF - *Umbilicus rupestris* flowers; URL - *Umbilicus rupestris* leaves. "-" No activity.

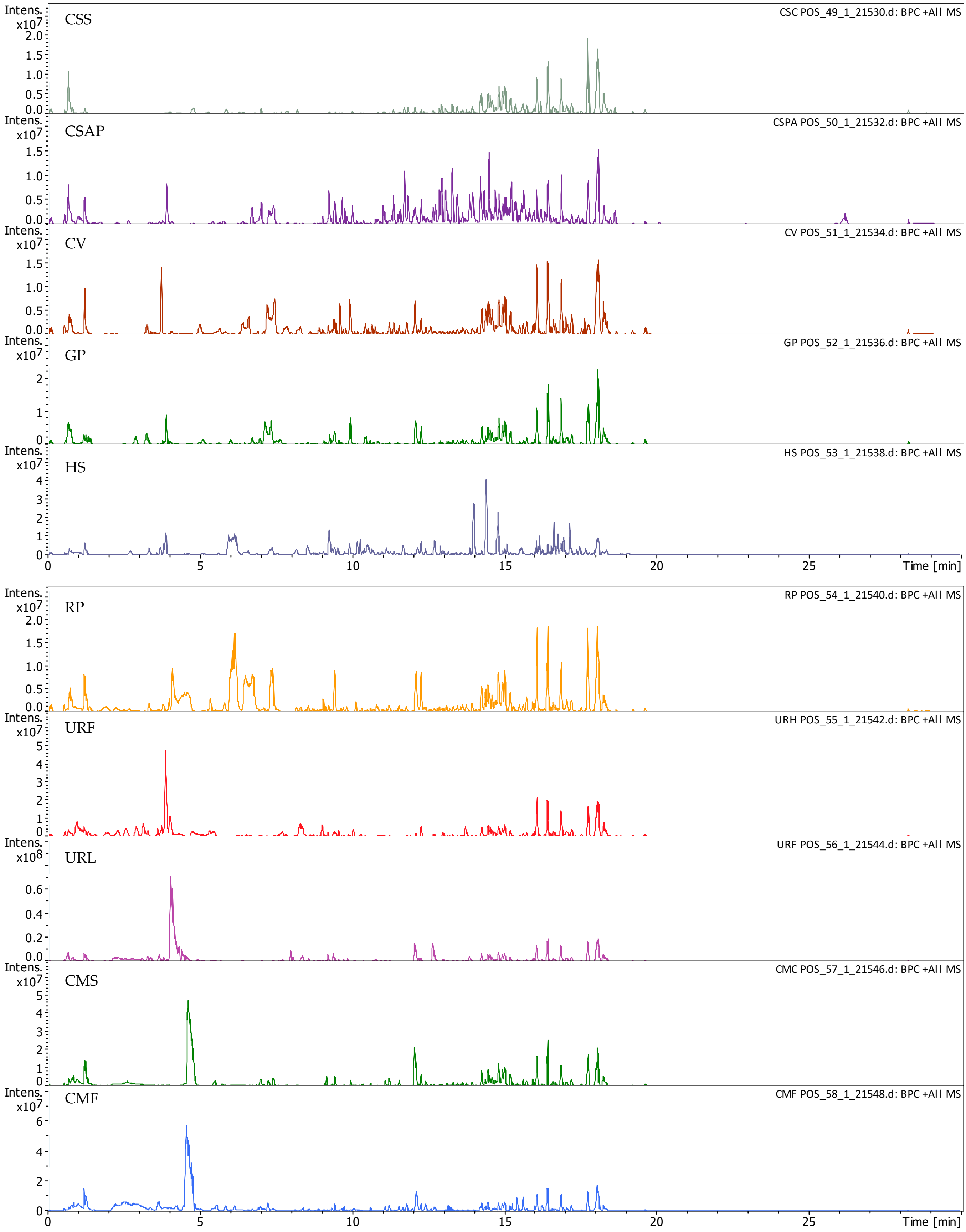


**Figure S1**. Base peak chromatogram (BPC) in positive ion mode of the analysed samples. *Cistus salviifolius* stems (CSS), *Cistus salviifolius* aerial parts (CSAP), *Clinopodium vulgare* (CV), *Glandora prostrata* (GP), *Helichrysum stoechas* (HS), *Rubia peregrina* (RP), *Umbilicus rupestris* flowers (URF), *Umbilicus rupestris* leaves (URL), *Coincya monensis* stems (CMS), and *Coincya monensis* flowers (CMF).

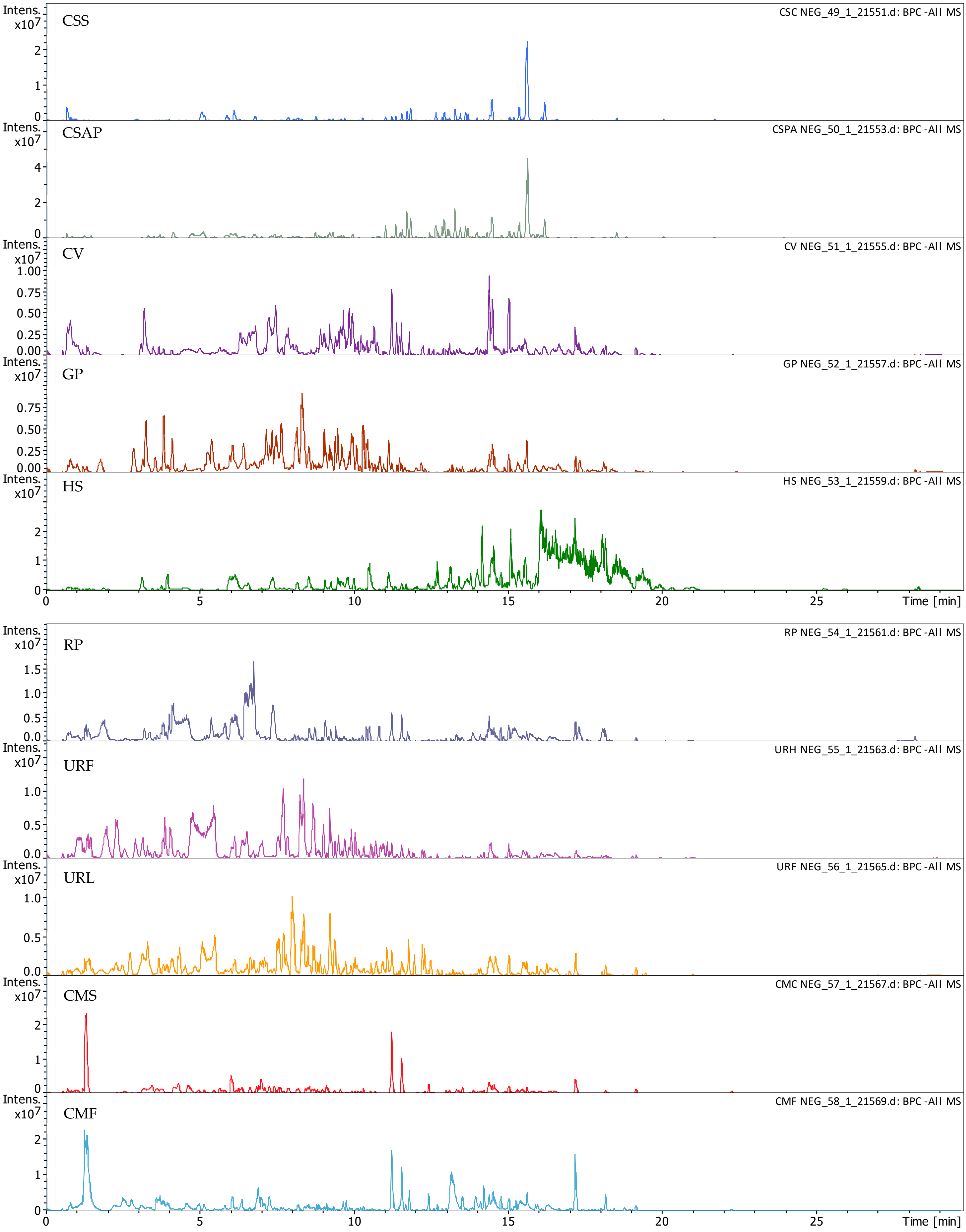


**Figure S2.** Base peak chromatogram (BPC) in negative ion mode of the analysed samples. *Cistus salviifolius* stems (CSS), *Cistus salviifolius* aerial parts (CSAP), *Clinopodium vulgare* (CV), *Glandora prostrata* (GP), *Helichrysum stoechas* (HS), *Rubia peregrina* (RP), *Umbilicus rupestris* flowers (URF), *Umbilicus rupestris* leaves (URL), *Coincya monensis* stems (CMS), and *Coincya monensis* flowers (CMF).
